# Traditional Chinese medicine interventions targeting Wnt/β-catenin signaling in cerebral ischemia/reperfusion injury: a review

**DOI:** 10.3389/fphar.2026.1902090

**Published:** 2026-08-04

**Authors:** Yijun Wu, Yongkang Sun, Yanbo Song, Xinzhi Wang

**Affiliations:** 1 Encephalopathy Center of The First Affiliated Hospital of Henan University of Chinese Medicine, Zhengzhou, China; 2 The First Clinical Medical College of Henan University of Chinese Medicine, Zhengzhou, China; 3 Henan Collaborative Innovation Center for Prevention and Treatment of Major Diseases by Integrated Traditional Chinese and Western Medicine, Zhengzhou, China; 4 Institute of Encephalopathy, Henan Academy of Chinese Medical Sciences, Zhengzhou, China

**Keywords:** acupuncture, cerebral ischemia/reperfusion injury, neuroprotection, review, traditional Chinese medicine, Wnt/β-catenin signaling

## Abstract

Vascular recanalization therapy for ischemic stroke commonly induces cerebral ischemia-reperfusion injury (CIRI), which causes secondary neural damage and substantially limits clinical benefit. The canonical Wnt/β-catenin signaling pathway has a central role in regulating central nervous system cell survival, vascular homeostasis, inflammatory balance, and neuroregeneration, making it a highly promising neuroprotective target for CIRI. Traditional Chinese medicine (TCM), including herbal-derived active compounds, compound formulas, and acupuncture, has distinctive multitarget regulatory advantages that are well aligned with the complex pathological features of CIRI. Existing basic studies indicate that TCM interventions can activate this pathway by inhibiting GSK-3β activity, stabilizing β-catenin, and promoting its nuclear translocation, thereby exerting multiple effects, including anti-apoptotic activity, blood-brain barrier (BBB) protection, anti-inflammatory and antioxidant actions, and promotion of neurogenesis and angiogenesis. This review systematically elucidates the molecular mechanisms by which Wnt/β-catenin signaling participates in CIRI-induced injury and repair, comprehensively summarizes the experimental evidence and action patterns of TCM interventions targeting this pathway for neuroprotection, and discusses the current limitations and future prospects for translating basic research into clinical application. This review aims to provide a solid theoretical basis for precision integrated Chinese-Western interventions in ischemic stroke and for the development of new neuroprotective agents.

## 1 Introduction

Ischemic stroke is one of the leading causes of death and long-term disability worldwide, and its disease burden remains characterized by high incidence, recurrence, and disability rates. Recent Global Burden of Disease studies have shown that stroke and its risk factors continue to impose substantial mortality, disability-adjusted life-year loss, and socioeconomic burden. In particular, against the background of population aging and the increasing prevalence of metabolic risk factors, stroke prevention and treatment remain a long-term challenge (Gbd 2021 Stroke Risk Factor Collaborators, 2024; Feigin et al., 2025). Reperfusion therapy is a key strategy for restoring blood flow, salvaging the ischemic penumbra, and improving prognosis during the acute phase of ischemic stroke. However, restoration of cerebral blood flow may itself induce cerebral ischemia/reperfusion injury (CIRI). CIRI is not a single pathological event, but a dynamic cascade driven by multiple interrelated processes, including impaired energy metabolism, oxidative stress burst, calcium overload, mitochondrial dysfunction, neuronal apoptosis, blood-brain barrier (BBB) disruption, amplification of neuroinflammation, and impaired remodeling of the neurovascular unit (Lan et al., 2024; Prabhakaran et al., 2026). Neuronal death largely determines the extent of early tissue injury, whereas BBB opening and inflammatory cell infiltration aggravate secondary cerebral edema and inflammatory amplification. By contrast, neurogenesis, angiogenesis, and synaptic remodeling influence later functional recovery. Therefore, elucidating the key molecular networks that govern the transition of CIRI from acute injury to chronic repair, and identifying intervention targets that can both suppress injury and promote repair, have become important directions in ischemic stroke research (Ma et al., 2021).

Wnt/β-catenin signaling is a canonical signal transduction pathway that regulates cell proliferation, differentiation, apoptosis, and tissue homeostasis (Maurice and Angers, 2025). Recent evidence suggests that dysregulation of this signaling pathway is broadly involved in the onset and progression of CIRI. Suppression of the pathway may promote neuronal apoptosis, aggravate BBB injury, and amplify neuroinflammatory responses, whereas appropriate activation is closely related to neurogenesis, angiogenesis, and restoration of neurovascular coupling (Huang X. et al., 2024; Yadava et al., 2025). Extensive crosstalk also exists between canonical and non-canonical Wnt branches and related signaling networks, including phosphoinositide 3-kinase/protein kinase B (PI3K/Akt), Notch, and microRNA (miRNA) pathways. Therefore, the Wnt/β-catenin pathway is not only an important regulatory node in the CIRI injury cascade, but may also serve as a central signaling hub linking early injury control with later repair and reconstruction.

Traditional Chinese medicine (TCM) has the characteristics of multitarget, holistic, and stage-specific intervention in the prevention and treatment of ischemic stroke. Previous studies have shown that multiple TCM interventions, including herbal formulas, Chinese patent medicines, herbal-derived active compounds, and acupuncture, may exert neuroprotective effects at several levels, such as attenuating oxidative stress, inhibiting inflammatory responses, protecting the BBB, regulating apoptosis, and promoting neurogenesis and angiogenesis. From the perspective of modern pharmacology, many plant-derived active compounds may participate in post-stroke injury repair by modulating the inflammatory microenvironment, redox homeostasis, and neurovascular unit function multitarget (Akhtar et al., 2026). Among these mechanisms, Wnt/β-catenin-related signaling has gradually been recognized as one of the important molecular pathways through which TCM may intervene in CIRI. However, the available evidence remains relatively scattered, and the stages of action, cellular targets, pathway branches, and upstream or downstream mechanistic links reported across different studies have not yet been systematically organized.

Based on this background, this review focuses on the mechanistic roles of Wnt/β-catenin signaling in CIRI and recent advances in its regulation by TCM. Specifically, it summarizes the regulatory roles of this pathway in key processes, including neuronal apoptosis, BBB homeostasis, neuroinflammation, oxidative stress and mitochondrial function, neurogenesis, and angiogenesis. It further reviews evidence that herbal formulas, Chinese patent medicines, herbal-derived active compounds, and acupuncture exert neuroprotective effects through Wnt/β-catenin-related signaling, with the aim of providing a reference for mechanistic research and precision TCM intervention in CIRI.

## 2 Wnt/β-catenin signaling pathway

Wnt/β-catenin signaling is an evolutionarily conserved cellular signal transduction network that contributes to embryonic development, tissue homeostasis, and regulation of central nervous system (CNS) function (Nusse and Clevers, 2017). From the perspective of signal transduction, the Wnt/β-catenin pathway is not a simple linear cascade, but a dynamic network involving multiple coordinated steps, including ligand secretion and modification, receptor complex assembly, formation of the Dishevelled (Dvl) signaling platform, inactivation of the destruction complex, β-catenin stabilization, and nuclear transcriptional regulation. Previous studies have systematically characterized proximal events such as Wnt ligand processing, Frizzled (FZD)/low-density lipoprotein receptor-related protein 5/6 (LRP5/6) receptor activation, Dvl polymerization, and membrane recruitment of Axin, indicating that the intensity, duration, and cell-specific effects of this pathway are regulated at multiple levels (Angers and Moon, 2009; MacDonald et al., 2009; Niehrs, 2012). Its core components include extracellular Wnt ligands, FZD transmembrane receptors, low-density LRP5/6 co-receptors, the intracellular signal hub Dvl, and the β-catenin destruction complex composed of glycogen synthase kinase-3β (GSK-3β), casein kinase 1α (CK1α), Axin, and adenomatous polyposis coli (APC) (Liu et al., 2022; Maurice and Angers, 2025). In the resting state, free cytoplasmic β-catenin is continuously captured and phosphorylated by the destruction complex and then degraded through the ubiquitin-proteasome pathway, keeping T-cell factor/lymphoid enhancer-binding factor (TCF/LEF)-associated target genes transcriptionally repressed (Wu et al., 2003; Daniels and Weis, 2005; Stamos and Weis, 2013). As a key scaffold protein in this complex, Axin recruits β-catenin and serine/threonine kinases, including CK1α and GSK-3β, into close spatial proximity, thereby facilitating sequential phosphorylation of β-catenin. CK1α is generally considered to initiate priming phosphorylation of β-catenin at Ser45, after which GSK-3β further catalyzes phosphorylation at Thr41, Ser37, and Ser33. The phosphodegron generated by phosphorylation at Ser33/Ser37 can be recognized by the E3 ubiquitin ligase β-transducin repeat-containing protein (β-TrCP), which subsequently mediates polyubiquitination of β-catenin and targets it for degradation by the 26S proteasome (Amit et al., 2002; Liu et al., 2002). Thus, in the absence of Wnt ligand stimulation, cytoplasmic β-catenin is usually maintained at a low level. Meanwhile, nuclear TCF/LEF transcription factors can bind Groucho/transducin-like enhancer of split (Groucho/TLE) co-repressors, maintaining the promoter regions of Wnt target genes in a relatively silent state (Cadigan and Waterman, 2012). When Wnt ligands bind to the receptor complex, Dvl is activated and Axin is recruited to the membrane receptor complex, thereby inhibiting the function of the β-catenin destruction complex. As a result, β-catenin escapes degradation, accumulates stably in the cytoplasm, and translocates into the nucleus (Wu et al., 2025; Xue et al., 2025). In the nucleus, β-catenin forms a complex with TCF/LEF and activates the transcription of target genes (Song et al., 2024). β-catenin has dual functions in cell adhesion and transcriptional coactivation, and its subcellular localization determines distinct biological effects. Membrane-associated β-catenin mainly contributes to the stability of cadherin-mediated cell-cell junctions, whereas cytoplasmic accumulation and nuclear translocation of β-catenin participate in transcriptional activation of Wnt target genes. This pathway critically regulates biological processes such as cell proliferation, differentiation, apoptosis, and tissue homeostasis. During development and tissue repair, Wnt signaling contributes to the maintenance of tissue homeostasis by regulating stem cell self-renewal, lineage differentiation, and regenerative programs. Its effects are highly context-dependent in time and space, and the same pathway may produce distinct or even opposing biological outcomes across different cell types, developmental stages, and pathological settings. Therefore, in the context of cerebral ischemia/reperfusion injury, interpretation of Wnt/β-catenin signaling should consider its dual regulatory properties, including its roles in promoting cell survival and regeneration as well as the potential consequences of aberrant activation (van Amerongen and Nusse, 2009; Valenta et al., 2012; Clevers et al., 2014). In addition to the canonical Wnt/β-catenin pathway, Wnt signaling can also act through β-catenin-independent branches, mainly including the Wnt/planar cell polarity (PCP) pathway and the Wnt/Ca^2+^ pathway (Inestrosa and Arenas, 2010). These branches share upstream components with the canonical pathway, such as Wnt ligands, Frizzled receptors, and Dishevelled, but differ in their downstream effects. The Wnt/PCP pathway mainly regulates cell polarity, cytoskeletal remodeling, and directional migration through molecules such as RhoA/Rac1-JNK, whereas the Wnt/Ca^2+^ pathway participates in calcium homeostasis, inflammatory responses, and immune regulation through effectors such as CaMKII, PKC, NFAT, and MAPK (Pascual et al., 2025).

During vascular development and BBB formation in the CNS, Wnt/β-catenin signaling exerts tissue-specific effects. Studies have shown that this pathway is particularly critical for CNS angiogenesis and establishment of the barrier phenotype, whereas its roles in non-CNS vessels are not entirely the same. These findings suggest that Wnt/β-catenin signaling is one of the core developmental programs through which brain vascular endothelial cells acquire barrier properties (Daneman et al., 2009; Reis and Liebner, 2013; Ben-Zvi and Liebner, 2022). In brain endothelial cells, ischemia/reperfusion injury can simultaneously downregulate canonical Wnt/β-catenin signaling and noncanonical Wnt/PCP and Wnt/Ca^2+^ signaling. Enhancing endothelial Wnt/β-catenin activity may help maintain tight junctions and BBB integrity, accompanied by restoration of noncanonical Wnt signaling (Ji et al., 2021b; Chen et al., 2026). In addition, context-dependent interactions exist between canonical and noncanonical Wnt signaling. For example, CaMKII can inhibit β-catenin/T-cell factor (TCF)-mediated transcriptional activity through the TAK1-NLK axis (Qin et al., 2024). Therefore, when evaluating Wnt-related effects in CIRI, β-catenin-dependent regulation should be distinguished from noncanonical Wnt-related effects.

In oncology, aberrant activation of Wnt/β-catenin signaling and nuclear accumulation of β-catenin are important drivers of malignant tumor proliferation, metastasis, and drug resistance (Song et al., 2024; Wu et al., 2025). In the cardiovascular system, the pathway shows biphasic regulatory characteristics, with protective effects in the acute phase and profibrotic and pathological hypertrophic effects in the chronic phase (Balatskyi et al., 2023). Its dysregulation is also closely associated with vascular smooth muscle cell phenotypic switching and atherosclerotic progression (Vallée, 2022).

**FIGURE 1:**
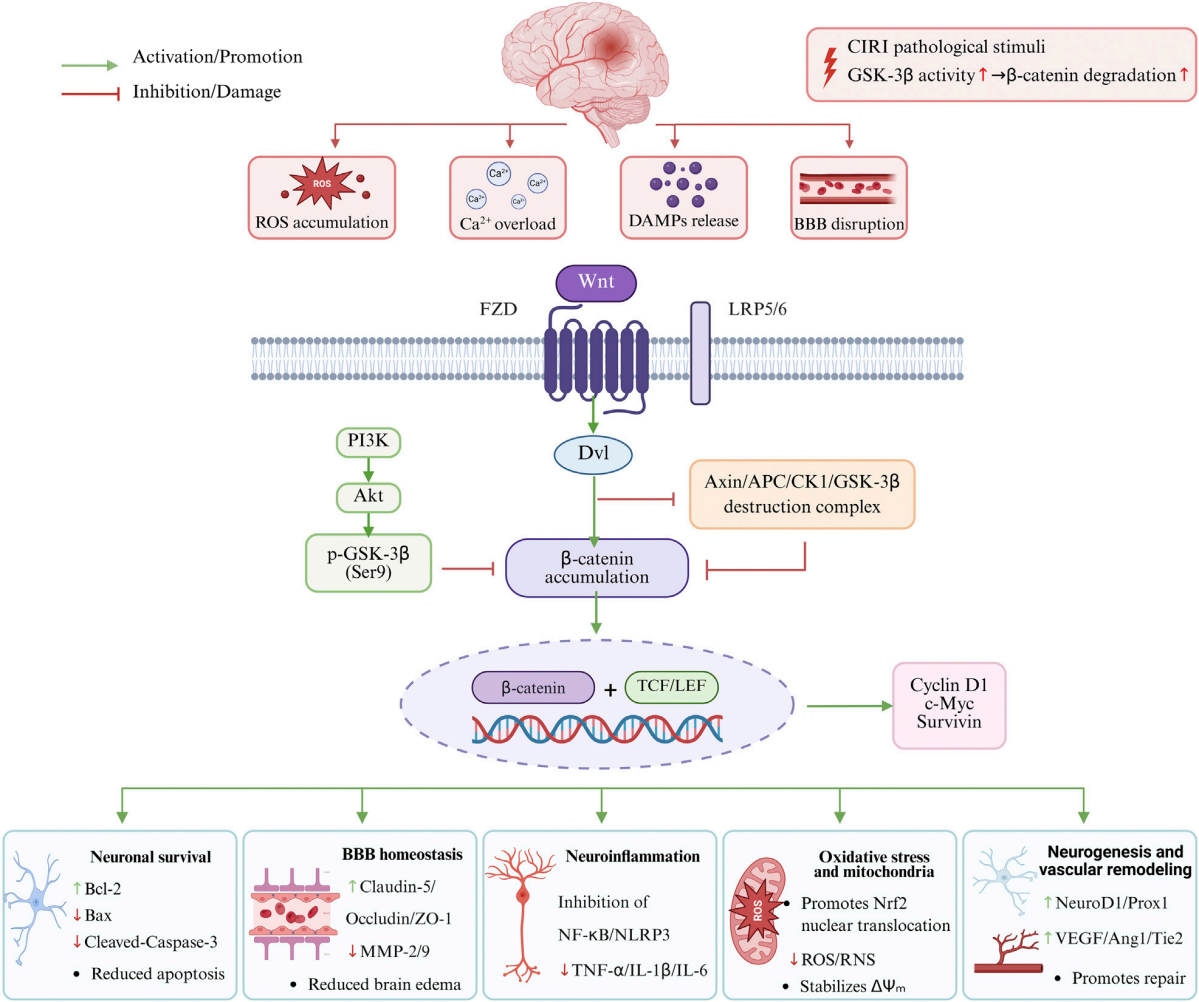
Mechanisms by which Wnt/β-catenin signaling participates in CIRI. Schematic illustration of the molecular mechanisms by which targeting the Wnt/β-catenin pathway ameliorates cerebral ischemia/reperfusion injury (CIRI). CIRI stimulation upregulates glycogen synthase kinase-3β (GSK-3β) activity and accelerates β-catenin degradation, thereby inducing multiple injury processes, including reactive oxygen species (ROS) accumulation and calcium overload. Activation of the Wnt pathway can inhibit the destruction complex, promote β-catenin accumulation and nuclear translocation, and regulate target gene expression, thereby protecting neurons, maintaining blood-brain barrier (BBB) homeostasis, suppressing inflammation and oxidative stress, and promoting neurovascular repair. Created in BioRender. Yang, W. (2026) https://BioRender.com/ip1maqv.

Accumulating evidence indicates that aberrant Wnt/β-catenin signaling is a shared pathological mechanism in various CNS diseases. Beyond regulation of the BBB, Wnt signaling is also involved in synapse formation, maintenance of neural circuits, and modulation of adult brain plasticity. Previous studies suggest that insufficient Wnt signaling can lead to striatal synaptic degeneration and abnormal motor behavior, whereas maintenance of BBB structure and function also depends on precise interactions among endothelial cells, pericytes, astrocytes, and the basement membrane. Together, these findings further indicate that Wnt/β-catenin-related networks are involved not only in CNS development, but also in adult brain barrier homeostasis, synaptic function, and post-injury repair (Britta and Stefan, 2014; Galli et al., 2014; Liebner et al., 2018). In CIRI, for example, neuronal apoptosis and BBB injury mediated by pathway imbalance are core pathological events, and activation of this signaling pathway is regarded as a neuroprotective strategy with considerable translational potential (Mo et al., 2022; Yadava et al., 2025). Abnormalities in this signaling pathway are also widely observed in Alzheimer disease, Parkinson disease, post-stroke cognitive impairment, multiple sclerosis, and traumatic brain injury, suggesting that it may serve as a common regulatory hub for CNS injury and repair (Tapia-Rojas and Inestrosa, 2018; Gao et al., 2021; Mo et al., 2022; Narvaes and Furini, 2022; Chi et al., 2023). Therefore, further clarification of Wnt/β-catenin regulatory mechanisms in CIRI may provide an important theoretical basis for precision TCM intervention in cerebral ischemia/reperfusion injury. The interventional role of this signaling pathway in CIRI is shown in (Figure 1).

## 3 Mechanistic roles of Wnt/β-catenin signaling in CIRI

CIRI is a secondary brain injury that occurs after blood flow is restored following the ischemic phase of stroke, and it represents a major pathological process contributing to neurological deficits. Its pathogenesis involves a cascade mediated by multiple factors, signaling pathways, and modes of cell death (Meng et al., 2025). Unlike ischemic injury alone, CIRI shows a typical time-dependent evolution and progressive amplification, broadly involving ischemic and reperfusion phases. During ischemia and hypoxia, impaired energy metabolism and insufficient ATP production disrupt ionic homeostasis, while excessive release of excitatory amino acids initiates neuronal injury. After abrupt restoration of blood flow and oxygen supply, more intense secondary injury may occur, characterized by ROS burst, severe oxidative stress, inflammatory cell infiltration, sustained release of inflammatory mediators, and activation of multiple programmed cell death pathways, including apoptosis, pyroptosis, and ferroptosis. These processes further impair the structure and function of the neurovascular unit, disrupt BBB integrity, and contribute to a pathological microenvironment that may amplify infarct development and neurological injury (Yang et al., 2025). This disordered microenvironment interferes with key biological processes such as neuronal survival, synaptic plasticity remodeling, BBB homeostasis, and neural repair. In parallel, the expression and function of several core signaling pathways become dysregulated, among which Wnt signaling is considered an important network regulating the balance between injury and repair in CIRI. Wnt signaling includes the canonical Wnt/β-catenin pathway and the non-canonical Wnt/PCP and Wnt/Ca^2+^ pathways, which may participate in CIRI through pathway-specific and context-dependent mechanisms (Ji et al., 2021b). Non-canonical Wnt branches are mainly involved in regulating brain cell polarity, cell migration, calcium homeostasis, cerebrovascular remodeling, and inflammatory responses, thereby indirectly contributing to brain injury regulation (Li L. et al., 2025). By contrast, the canonical Wnt/β-catenin pathway is closely associated with CIRI progression, neuroprotection, and neural repair, and abnormal changes in its activity may influence the severity of brain injury and subsequent recovery.

**FIGURE 2:**
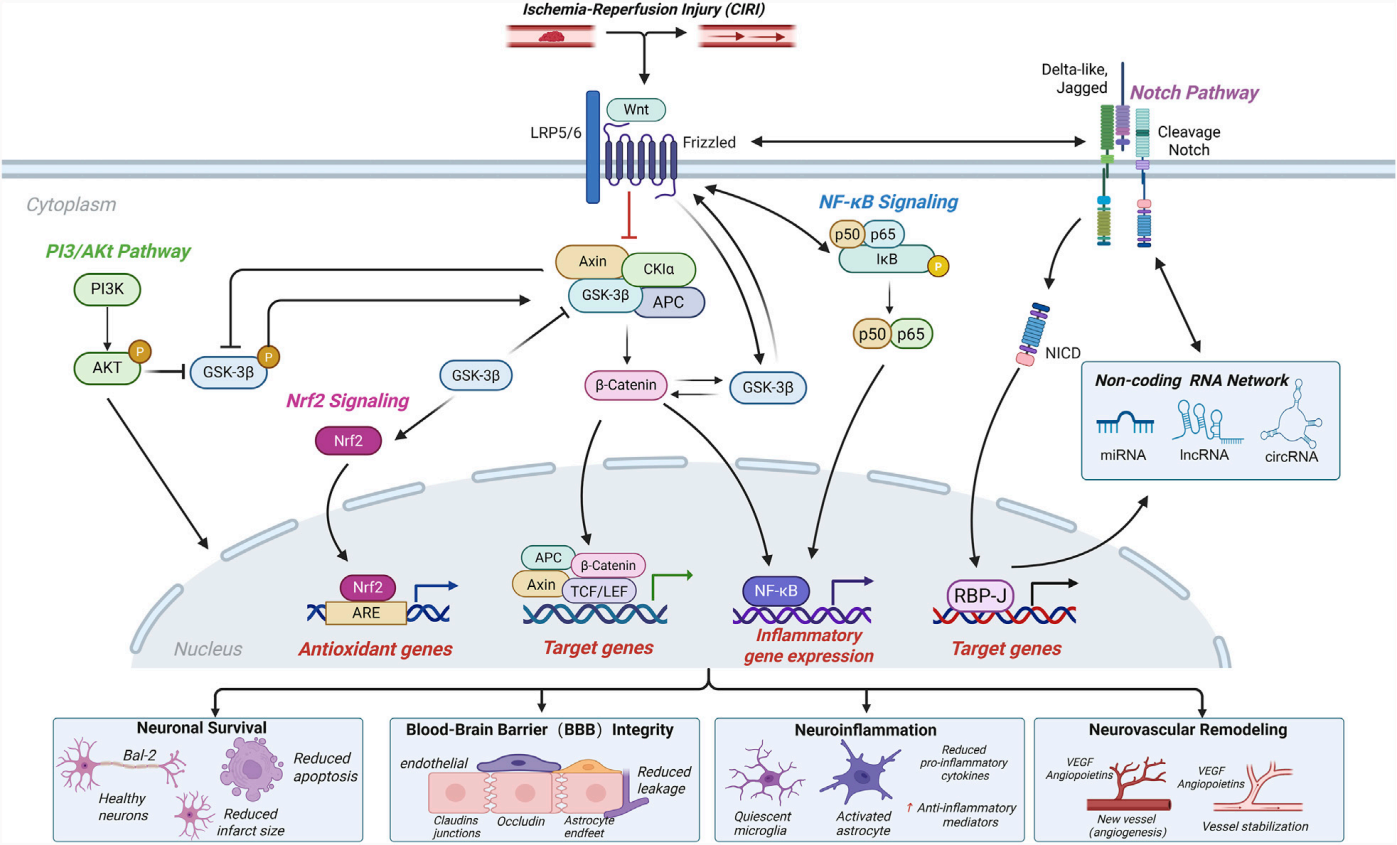
Wnt/β-catenin signaling as a central regulatory hub in cerebral ischemia/reperfusion injury. The schematic illustrates the interactions between Wnt/β-catenin signaling and PI3K/Akt, Nrf2, NF-κB, Notch, and non-coding RNA networks, and summarizes their downstream effects on neuronal survival, BBB integrity, neuroinflammation, and neurovascular remodeling. Created in BioRender. Yang, W. (2026) https://BioRender.com/1swptdl.

Functional suppression of the canonical Wnt/β-catenin pathway appears to be an important molecular mechanism contributing to CIRI progression. Studies based on MCAO/R animal models have shown that, after CIRI, the expression levels of Wnt3a and β-catenin in ischemic brain tissue are significantly decreased, and these changes are associated with increased infarct volume, greater neuronal apoptosis, and more severe neurological deficits (Zhang et al., 2019; Geng et al., 2025). At the molecular level, CIRI may abnormally enhance GSK-3β activity. Activated GSK-3β promotes phosphorylation of β-catenin and facilitates its degradation through the ubiquitin pathway, thereby preventing stable cytoplasmic accumulation of β-catenin and impairing its nuclear translocation (Matei et al., 2018). This process reduces the activity of the β-catenin/TCF/LEF transcriptional complex and suppresses downstream target genes involved in neuroprotection, anti-apoptotic regulation, BBB structural maintenance, and neural regeneration and repair, ultimately aggravating neuronal injury and pathological brain damage (Wang W. et al., 2017). Taken together, dysregulation of the Wnt/β-catenin pathway may represent a key event in the CIRI pathological cascade and an important target for modulating the balance between post-reperfusion neural injury and repair. The integrated regulatory network centered on Wnt/β-catenin signaling in CIRI is summarized in (Figure 2).

### 3.1 Regulation of neuronal apoptosis and cell survival

Neuronal apoptosis is an important pathological basis of neurological deficits after CIRI. Energy metabolism disorders after cerebral ischemia cause mitochondrial dysfunction, accumulation of reactive oxygen species (ROS), and intracellular Ca^2+^ overload, thereby activating the caspase cascade and inducing programmed neuronal death. Canonical Wnt/β-catenin signaling is an important endogenous protective mechanism regulating neuronal survival. Under physiological conditions, binding of Wnt ligands to FZD receptors and LRP5/6 co-receptors recruits Dvl and inhibits the activity of the β-catenin destruction complex composed of Axin, APC, CK1, and GSK-3β. This allows β-catenin to accumulate in the cytoplasm and translocate into the nucleus. Nuclear β-catenin then binds to TCF/LEF transcription factors and initiates transcription of target genes such as Cyclin D1, c-Myc, and Survivin, thereby enhancing cell survival and inhibiting apoptosis (Chen et al., 2020; Mo et al., 2022; Anand et al., 2023; Yadava et al., 2025). After cerebral ischemia, abnormally increased GSK-3β activity promotes β-catenin phosphorylation and degradation, blocks its nuclear translocation, and reduces the expression of downstream anti-apoptotic genes (Wang W. et al., 2017). Studies show that activation of Wnt/β-catenin signaling can increase B-cell lymphoma 2 (Bcl-2) expression and suppress Bcl-2-associated X protein (Bax) and cleaved caspase-3 levels, thereby maintaining mitochondrial membrane stability and reducing neuronal apoptosis (Li et al., 2023). In addition, Wnt/β-catenin signaling interacts with the PI3K/Akt pathway. Activated Akt can inhibit GSK-3β activity through phosphorylation at Ser9, reducing β-catenin degradation, whereas β-catenin can in turn enhance the expression of cell survival-related genes. Together, these interactions form a PI3K/Akt-GSK-3β-β-catenin neuroprotective axis (Gerace et al., 2020).

### 3.2 Maintenance of BBB homeostasis and cerebrovascular function

BBB disruption is an important pathological feature distinguishing CIRI from cerebral ischemia alone. After reperfusion, opening of the BBB, which is composed of brain microvascular endothelial cells, pericytes, astrocytic end-feet, and the basement membrane, leads to plasma protein extravasation, inflammatory cell infiltration, and cerebral edema, thereby contributing to secondary brain injury (Mo et al., 2022; Anand et al., 2023; Yadava et al., 2025). Wnt/β-catenin signaling is a core signaling axis for BBB formation and maintenance. During brain development, Wnt7a and Wnt7b secreted by neural tissue activate β-catenin signaling in brain microvascular endothelial cells through the G protein-coupled receptor 124 (GPR124)/reversion-inducing cysteine-rich protein with Kazal motifs (RECK) co-receptor system, inducing brain-specific angiogenesis and BBB maturation. In adult brain tissue, this signaling pathway continues to participate in the maintenance of cerebrovascular homeostasis (Ji et al., 2021a). After ischemia/reperfusion, β-catenin expression in brain microvascular endothelial cells decreases markedly, leading to reduced transcription of tight junction proteins such as Claudin-5, Occludin, and zonula occludens-1 (ZO-1) (Manukjan et al., 2024). Meanwhile, activation of matrix metalloproteinase-2 (MMP-2) and MMP-9 causes basement membrane degradation and vascular integrity damage, further increasing BBB permeability (Chen et al., 2021). Restoration of Wnt/β-catenin activity can increase tight junction protein expression, inhibit MMP-mediated extracellular matrix degradation, and thereby reduce the risk of cerebral edema and hemorrhagic transformation. In models of ischemic brain injury, enhancement of β-catenin-related signaling may also alleviate BBB disruption by upregulating tight junction proteins such as Claudin-5, Occludin, and ZO-1, thereby reducing endothelial cell injury and increased vascular permeability. Recent studies further suggest that the Wnt/β-catenin/CYP1B1 axis can attenuate oxidative stress and protect the BBB under CIRI conditions, while β-catenin-mediated protection of brain endothelial cells may also contribute to the ability of certain metabolic drugs to ameliorate pre-ischemic BBB injury (Chen et al., 2024; Liu G. et al., 2025).

### 3.3 Regulation of neuroinflammation

Neuroinflammation occurs throughout the entire course of CIRI and is an important driver of secondary neuronal injury. After ischemia, the release of damage-associated molecular patterns (DAMPs) activates microglia, astrocytes, and the peripheral immune system, generating a persistent inflammatory microenvironment. The Wnt/β-catenin pathway is a key regulatory mechanism for maintaining CNS immune homeostasis. It participates in regulation of the post-ischemic inflammatory microenvironment through two independent routes. First, β-catenin can competitively antagonize the nuclear factor-κB (NF-κB) pathway, blocking NF-κB-mediated transcription of proinflammatory genes and downregulating tumor necrosis factor-α (TNF-α), interleukin-1β (IL-1β), IL-6, cyclooxygenase-2 (COX-2), and other proinflammatory molecules. Second, intact activation of Wnt signaling can regulate microglial polarization by promoting differentiation toward the anti-inflammatory reparative M2 phenotype while inhibiting transformation toward the proinflammatory damaging M1 phenotype. These dual regulatory effects improve the post-stroke inflammatory microenvironment in the brain and ultimately mediate neuroprotection (Liu R.-J. et al., 2025). In addition, the NOD-like receptor family pyrin domain-containing 3 (NLRP3) inflammasome is an important effector of neuroinflammation after cerebral ischemia (Wang et al., 2022). ROS accumulation promotes NLRP3 inflammasome assembly and activates caspase-1, resulting in the maturation and release of IL-1β and IL-18. Studies have found that restoration of β-catenin expression can inhibit excessive NLRP3 activation and thereby attenuate inflammatory cascades (Chen B. et al., 2022).

### 3.4 Regulation of oxidative stress and mitochondrial homeostasis

Oxidative stress bursts and mitochondrial dysfunction are core pathological events in CIRI. After restoration of blood flow, excessive accumulation of ROS and reactive nitrogen species (RNS) induces lipid peroxidation of cell membranes, oxidative modification of proteins, and genomic DNA damage. These events further impair mitochondrial structural integrity and energy metabolism homeostasis, producing a cascade of neuronal injury (Wu et al., 2020). Recent studies suggest close crosstalk between Wnt/β-catenin signaling and the nuclear factor erythroid 2-related factor 2 (Nrf2) antioxidant axis, which constitutes an important endogenous regulatory pathway against oxidative injury in CIRI. After pathway activation, nuclear β-catenin can promote Nrf2 nuclear translocation and transcriptionally upregulate antioxidant enzymes such as heme oxygenase-1 (HO-1), NAD(P)H quinone oxidoreductase 1 (NQO1), and superoxide dismutase (SOD), thereby clearing excessive free radicals and maintaining cellular redox homeostasis (Cui et al., 2021). Conversely, excessive activation of GSK-3β in CIRI not only promotes β-catenin ubiquitination and degradation, thereby suppressing Wnt signaling activity, but also accelerates proteasomal degradation of Nrf2 and reduces endogenous antioxidant capacity. This creates a vicious cycle between oxidative stress injury and inactivation of the Wnt/β-catenin/Nrf2 pathway (Wu et al., 2017).

In addition to cooperating with Nrf2 to antagonize oxidative stress, activated Wnt/β-catenin signaling can also regulate mitochondrial homeostasis. On the one hand, it improves mitochondrial structure and energy metabolism by inhibiting abnormal mitochondrial fission, stabilizing mitochondrial membrane potential, and blocking pathological opening of the mitochondrial permeability transition pore (mPTP) (Cui et al., 2021). On the other hand, it regulates the expression of key mitochondrial dynamics molecules, including mitofusin 2 (Mfn2), optic atrophy 1 (OPA1), and dynamin-related protein 1 (Drp1), maintaining the dynamic balance between mitochondrial fusion and fission and improving adenosine triphosphate (ATP) synthesis efficiency. Through both antioxidant and mitochondrial protective mechanisms, Wnt/β-catenin signaling may attenuate neuronal injury caused by CIRI (Huang Y. et al., 2024).

### 3.5 Promotion of neurogenesis and neurovascular unit remodeling

Wnt/β-catenin signaling is an important driver of neurogenesis and angiogenesis (Knotek et al., 2020; Li W. et al., 2025). After ischemic stimulation, neural stem cells in the subventricular zone (SVZ) and hippocampal dentate gyrus are activated. After β-catenin enters the nucleus, it can induce the expression of genes such as Cyclin D1, NeuroD1, and Prox1, thereby promoting proliferation, migration, and neuronal differentiation of neural precursor cells (Kriska et al., 2021). At the same time, Wnt/β-catenin signaling is involved in the regulation of angiogenesis. β-Catenin can promote the expression of vascular endothelial growth factor (VEGF), angiopoietin-1 (Ang1), and tyrosine kinase with immunoglobulin-like and EGF-like domains 2 (Tie2), thereby enhancing endothelial cell migration and vascular formation (Khamchai et al., 2022). According to the currently proposed theory of neurovascular regeneration coupling, neurogenesis and angiogenesis cooperate to complete tissue repair under shared signaling networks, and Wnt/β-catenin is an important coordinator of this process.

### 3.6 Regulation of non-coding RNA networks

The pathological progression of CIRI is regulated by complex epigenetic mechanisms, in which ncRNAs represent important regulatory molecules acting at transcriptional and post-transcriptional levels without encoding proteins (Yang et al., 2022). Wnt/β-catenin signaling is essential for maintaining CNS homeostasis, and its activation status is closely associated with the severity of CIRI and subsequent outcomes. Existing evidence indicates that ischemia-hypoxia, oxidative stress, and neuroinflammation induced by CIRI can markedly reshape ncRNA expression profiles (Su et al., 2022). Different ncRNA subtypes may target core components of Wnt/β-catenin signaling and thereby participate in key pathological processes, including neuronal injury, glial activation, BBB disruption, and endogenous neurogenesis, forming a multidimensional epigenetic regulatory network in CIRI.

miRNAs are among the most extensively studied ncRNA subtypes involved in Wnt/β-catenin regulation. By binding to mRNAs of key pathway-related genes through sequence complementarity, miRNAs can modulate pathway activity through gene silencing or degradation, thereby influencing the balance between injury and repair in CIRI. Functional studies have shown that miR-148b targets and suppresses Wnt-1 expression, inhibits Wnt pathway activation, reduces neural stem cell proliferation and differentiation, and impairs endogenous brain repair; silencing this miRNA could attenuate or reverse these detrimental effects (Wang J. et al., 2017). In addition, inhibition of miR-155 has been reported to activate Wnt/β-catenin signaling, correct the imbalance between T helper 17 (Th17) and regulatory T (Treg) cells after CIRI, reduce excessive CNS inflammation, and alleviate ischemic brain injury (Huang et al., 2025). These findings suggest that aberrant miRNA expression may contribute to disruption of the homeostatic balance between neural injury and repair in CIRI.

lncRNAs, circRNAs, and exosome-derived ncRNAs may also participate in CIRI regulation by indirectly modulating Wnt/β-catenin signaling activation (Gao et al., 2020; Wang et al., 2020). Mechanistic studies suggest that lncRNA NEAT1 can enhance Wnt3a mRNA stability and increase Wnt3a protein expression, thereby activating Wnt signaling, suppressing excessive microglial activation and neuronal apoptosis, and exerting neuroprotective effects. This protection was reported to be abolished or reversed by a specific Wnt pathway inhibitor (Zhou et al., 2022). circCTNNB1 may alleviate CIRI by acting through a miRNA sponge mechanism to sequester miR-96-5p and upregulate scavenger receptor class B type 1 (SR-B1) expression, although its effects on nuclear translocation of β-catenin and downstream transcriptional activity require further validation (Chen C. et al., 2022). In addition, miR-124 enriched in M2 microglia-derived exosomes may mediate intercellular communication in the brain, suppress inflammatory cascades, and help restore neurovascular unit homeostasis, providing a potential direction for noninvasive targeted intervention in CIRI (Song et al., 2019).

Overall, the ncRNA-Wnt/β-catenin regulatory network may constitute an important epigenetic mechanism involved in the fine regulation of CIRI pathology. Further clarification of this network may help identify promising targets for precision prevention, treatment, and targeted drug development in ischemic stroke.

## 4 Regulation of Wnt/β-catenin signaling by TCM interventions in CIRI

As shown in (Table 1), a broad range of TCM interventions has been investigated for their ability to regulate Wnt/β-catenin-related signaling in experimental models of CIRI. These interventions range from herbal-derived active compounds to complex herbal formulas, Chinese patent medicines, and acupuncture-based therapies. Their reported effects include inhibition of neuronal apoptosis and neuroinflammation, preservation of BBB integrity, promotion of endogenous neurogenesis and angiogenesis, and support of post-ischemic white matter repair. Together, these studies provide a mechanistic basis for understanding how TCM may intervene in CIRI through Wnt/β-catenin-centered regulatory networks.

**TABLE 1:** Experimental studies of TCM interventions regulating Wnt/β-catenin-related signaling in CIRI.

Category	Intervention	Model/system	*In vivo*/*in vitro*	Treatment regimen	Molecular targets	Mechanism/effects	References
Active compound Phenolic acids/phenylpropanoids	Salvianolic acid A (SAA)	Rat ischemic stroke model induced by electrocoagulation-generated autologous thrombus; SVZ neural stem/progenitor cell-related assays	*In vivo*/*in vitro*	10 mg/kg by gavage for 14 days	Wnt3a↑; p-GSK-3β↑; β-catenin↑; TCF-4, Cyclin D1, NeuroD1, and BDNF↑; Bcl-2/Bax↑; cleaved caspase-3/9↓	Promotes endogenous neurogenesis and axonal regeneration; inhibits neuronal apoptosis	Zhang et al. (2022)
Active compound Phenolic acids/phenylpropanoids	p-Hydroxybenzaldehyde (p-HBA)	Rat tMCAO model; reactive astrocyte-to-neuron transdifferentiation-related assays	*In vivo*/*in vitro*	Administered during the acute phase of stroke; dose not stated in the source text	Wnt3a↑; p-GSK-3β↑; β-catenin↑	Promotes astrocyte-to-neuron transdifferentiation, induces angiogenesis, and preserves the neurovascular unit	Yuan et al. (2022)
Active compound Phenylethanoid glycosides	Phenylethanoid glycosides (PhGs)	SVZ neural stem cell OGD/R model; mouse MCAO model	*In vivo*/*in vitro*	15 and 30 μg/mL; 100 and 200 mg/kg by gavage for 7 days	Wnt3a↑; total/nuclear β-catenin↑; GSK-3β↓; c-Myc↑; effects blocked by IWR-1	Activates the canonical Wnt pathway and promotes neural stem cell proliferation and endogenous neuroregeneration	Liu et al. (2023)
Active compound Flavonoids	Galangin	Rat MCAO model	*In vivo*	Not stated in the source text	Wnt/β-catenin-HIF-1α/VEGF signaling; β-catenin and VEGF as key nodes	Protects the neurovascular unit and BBB integrity; promotes angiogenesis and tissue repair	Wu et al. (2015)
Active compound Flavonoids	Naringenin (NAR)	Mouse MCAO/R model	*In vivo*	5, 10, and 20 mg/kg; 10 mg/kg identified as an optimal dose; observation up to 7 days after reperfusion	p-GSK-3β(Ser9)↑; β-catenin↑; ZO-1, Occludin, and Claudin-5↑; IL-1β, IL-6, and TNF-α↓	Protects the BBB, reduces evans blue leakage, suppresses inflammation and apoptosis, and attenuates ischemic penumbra injury	Yang et al. (2024)
Active compound Related polyphenols	Curcumin	Mouse global cerebral ischemia model induced by bilateral common carotid artery occlusion; hippocampal neural stem cell-related assays	*In vivo*/*in vitro*	50 and 100 mg/kg/day	Wnt3a↑; β-catenin↑; p-GSK-3β/GSK-3β↑; Ngn2, Pax6, and NeuroD1↑; effects blocked by DKK1	Promotes hippocampal dentate gyrus neurogenesis and improves spatial learning and memory deficits	Yang et al. (2021b)
Active compound Flavonoids/isoflavonoids	Medicarpin	Mouse cerebral ischemia/reperfusion model; BV2 microglial and N2A neuronal models	*In vivo*/*in vitro*	0.5 and 1.0 mg/kg	PI3K/Akt↑; GSK-3 inactivation; β-catenin accumulation; p65 NF-κB↓; activated caspase-3↓; DCX, BDNF, TrkB, and Bcl-2↑; effects blocked by LY294002	Suppresses neuroinflammation and neuronal apoptosis, induces endogenous neurogenesis, and protects the BBB	Chern et al. (2021)
Active compound Terpenoids/lactones	L-borneolum/D-borneolum	Rat tMCAO ischemia/reperfusion model	*In vivo*	50–200 mg/kg by continuous gavage	L-borneolum: Wnt3a↓ and Notch-1↓; D-borneolum: GSK-3β/β-catenin↑; VEGF-independent effects	Improves cerebral microcirculation and promotes striatal astrocyte conversion into mature GABAergic neurons or Nestin + neural stem cells	Ma et al. (2023)
Active compound Terpenoids/lactones	Senkyunolide I (SEI)	Embryonic rat-derived neural stem/progenitor cells	*In vitro*	200 and 400 μM	Akt phosphorylation↑; active β-catenin↑; effects reversed by LY294002; no obvious change in GSK-3β Ser9 phosphorylation	Promotes neural stem/progenitor cell proliferation through the Akt/β-catenin pathway and may expand the stem-cell pool	Wang et al. (2023)
Active compound Terpenoids/triterpenoids	Ilexonin A (IA)	Rat MCAO/R model; neuroregeneration assessment in peri-ischemic tissue	*In* *vivo*	40 mg/kg intraperitoneally; administered immediately after reperfusion and then once daily; assessed at 1, 3, 7, and 14 days	β-catenin protein↑; LEF1 mRNA↑; GSK-3β protein↓; Axin mRNA↓; BrdU/nestin and BrdU/NeuN double-positive cells↑	Activates the canonical Wnt pathway, promotes post-ischemic neuronal proliferation, differentiation, and neuroregeneration, and alleviates neurological deficits	Zhang et al. (2016)
Active compound Saponins	Ginsenoside Rb1 (G-Rb1)	BV2 microglial OGD/R model; C57BL/6J mouse MCAO/R model	*In vivo*/*in vitro*	10 μM *in vitro*; 40 mg/kg intraperitoneally once daily for 3 days before modeling; XAV939 at 30 μM or 5 mg/kg; assessed at 1, 3, 7, and 14 days after surgery	β-catenin↑; GSK-3β↓; M2 microglial proportion↑; Arg-1 and CD206↑; TNF-α and IL-1β↓; effects reversed by XAV939	Activates Wnt/β-catenin signaling, regulates microglial M1/M2 polarization, attenuates neuroinflammation, restores cerebral blood flow, and reduces infarct volume	Lu et al. (2025)
Active compound Saponins	Astragaloside IV (As IV)	Photothrombotic mouse ischemic stroke model; embryonic hippocampal neural stem cell culture system	*In vivo*/*in vitro*	2 mg/kg intravenously; 10 and 100 nM	IL-17↓; Wnt2↑; β-catenin↑; GSK-3β↓; DCX, BrdU, and Sox2↑	Inhibits IL-17 and activates Wnt signaling; promotes hippocampal neurogenesis and improves cognitive deficits	Sun et al. (2020)
Active compound Saponins	Total saponins of trillium tschonoskii (TSTT)	Rat permanent MCAO model	*In vivo*	33 and 65 mg/kg	p-GSK-3β(Ser9)↑; p-β-catenin↓; p-CRMP-2↓; Ki67/NG2 and Ki67/CNPase↑	Regulates the GSK-3/β-catenin/CRMP-2 axis and promotes oligodendrogenesis, remyelination, axonal remodeling, and white matter repair	Yang et al. (2021a)
Herbal extract Other category	Mallotus oblongifolius (MO) extract	Mouse CIRI model induced by bilateral common carotid artery ligation; C17.2 neural stem cell OGD/R model	*In vivo*/*in vitro*	*In vivo*: 0.57, 1.70, and 5.10 g/kg/day, calculated as crude leaf material; behavioral assessment on days 4, 7, and 10; *in vitro*: 0.18, 0.53, and 1.60 mg/mL for 48 h	β-catenin↑; Cyclin D1↑; nestin↑; EdU-positive cells↑	Activates the Wnt/β-catenin/Cyclin D1 axis, promotes *in situ* NSC proliferation, and improves motor, sensory, balance, and grasping abilities	Li et al. (2020)
Active compound Other category	Hirudin	BMEC OGD/R model; rat MCAO/R model	*In vivo*/*in* *vitro*	20, 40, and 80 μg/mL; 40 mg/kg intraperitoneally for 7 days	Wnt3a↑; β-catenin↑; CD34, VEGF, and Ang-2↑; DKK1 reverses the pro-angiogenic effect	Promotes angiogenesis in the ischemic penumbra and reduces neurological deficit scores and infarct volume	He et al. (2025)
Active compound Other category	Echinacoside (ECH)	PC12 cell OGD/R model; mouse MCAO model; CK2α′ heterozygous knockout mice	*In vivo*/*in vitro*	2.5–10 μmol/L; *in vivo* dose not stated in the source text	Covalent binding to CK2α′ K171; BTF3α complex formation; Wnt/β-catenin↑; β-catenin nuclear translocation↑; Mfn2↑	Promotes mitochondrial fusion, maintains mitochondrial membrane potential and mtDNA homeostasis, and attenuates ischemic brain injury	Zeng et al. (2021), Zeng et al. (2025)
Herbal formula/patent preparation	Danggui-Jakyak-San (DJS)	Mouse transient forebrain ischemia model induced by bilateral common carotid artery occlusion	*In vivo*	Delayed long-term administration; dose not stated in the source text	p-Akt↑; p-GSK-3β↑; β-catenin↑	Activates the Akt/GSK-3β/β-catenin axis, promotes hippocampal dentate gyrus neurogenesis, and improves spatial memory impairment	Song et al. (2013)
Herbal formula/patent preparation	Fuzheng Jiedu Tongluo Granule (FZJDTL)	Rat MCAO/R model; OGD/R-injured bEnd.3 endothelial cells	*In vivo*/*in vitro*	3.24, 6.48, and 12.96 g/kg by gavage once daily for 7 days; 1%–20% drug-containing serum; XAV939 at 40 mg/kg	Wnt3a↑; β-catenin↑; Mfsd2a↑; claudin-5↑; Cav-1↓; Cav-1-mediated transcytotic vesicles↓; EB/albumin/SF leakage↓; TEER↑	Activates the Wnt/β-catenin/Mfsd2a/Cav-1 axis, inhibits cerebrovascular endothelial transcytosis, protects BBB function, and alleviates CIRI	Liu et al. (2026)
Herbal formula/patent preparation	Danhong injection (DHI)	High-fat diet combined with rat MCAO/R model	*In vivo*	1.0 and 2.0 mL/kg via tail-vein injection for 7 days; MCAO/R on day 8 with additional administration 6 h after reperfusion; DKK1 at 0.1 mg/mL, 10 μL, by intracerebroventricular injection	Wnt3a↑; p-GSK-3β↑; nuclear β-catenin↑; Cyclin D1↑; Claudin-5, Occludin, and ZO-1↑; MMP-9↓; NeuN + cells↑; IgG leakage↓; effects reversed by DKK1	Activates Wnt/β-catenin signaling, enhances tight junction proteins, inhibits MMP-9, preserves BBB integrity under hyperlipidemic conditions, and attenuates infarction and cortical injury	Shi et al. (2026)
Herbal formula/patent preparation	Yangyin Yiqi Huoxue prescription (YYH)	Rat MCAO model; BV2/HT22 OGD/R and co-culture models	*In vivo*/*in vitro*	0.83, 1.65, and 3.3 g/kg; 10% drug-containing serum	Wnt3a↑; β-catenin↑; GSK-3β↓; Wnt5a↓; IL-1β, IL-6, and TNF-α↓; NLRP3↓; effects reversed by DKK1	Activates the canonical Wnt pathway and suppresses the non-canonical Wnt5a pathway; promotes neural stem cell proliferation/differentiation and inhibits microglia-mediated inflammation	Lu et al. (2025)
Herbal formula/patent preparation	Huang-Lian-Jie-Du Decoction (HLJDT)	Rat MCAO cerebral ischemia/reperfusion model	*In vivo*	0.9 g/kg	Wnt7a/β-catenin↑; Occludin, E-cadherin, and Mfsd2a↑; Cav-1↓; IFN-γ and IL-17A↓; IL-4 and TGF-β↑; LFA-1/ICAM-1↓	Repairs transcellular and paracellular BBB pathways, blocks central CD4^+^ T-cell infiltration, and attenuates neuroimmune inflammation	Chen et al. (2025)
Herbal formula/patent preparation	Buyang Huanwu Decoction (BHD)	Mouse MCAO model; Cav1 knockout mice	*In vivo*	18.5 g/kg	Cav1↑; Wnt1↑; β-catenin↑; GSK3β↓; BDNF, NGF, and GDNF↑	Promotes hippocampal neural stem cell proliferation, newborn neuron differentiation/maturation, and endogenous neuroregeneration through the Cav1-Wnt/β-catenin axis	OuYang et al. (2025)
Herbal formula/patent preparation	Gujin Luyan Xuming Decoction (XMD)	Rat MCAO/R model; bEnd.3 endothelial cell OGD/R model	*In vivo*/*in vitro*	10.26, 20.52, and 41.04 g/kg/day; 10% drug-containing serum	RAB11A↑; Wnt5a↑; β-catenin↑; VEGFA↑; VEGFR2↑	Activates the RAB11A-mediated Wnt5a/β-catenin/VEGFA-VEGFR2 axis, promotes angiogenesis, and attenuates ischemic brain injury	Cai et al. (2026)
Herbal formula/patent preparation	Dandeng Tongnao Capsules	Rat MCAO model; OGD/R-injured BMECs	*In vivo*/*in vitro*	1%–20% drug-containing cerebrospinal fluid; *in vivo* dose not stated in the source text	Wnt-1↑; β-catenin↑; LRP-6↑; GSK-3β↓; Bax↓; Bcl-2↑	Activates the Wnt-1/LRP-6/β-catenin pathway, attenuates cerebral microvascular endothelial injury, and protects the BBB	Huang et al. (2022)
Acupuncture/electroacupuncture	Scalp electroacupuncture (Baihui, GV20)	Rat MCAO/R model	*In vivo*	30 min/day for 7, 14, or 21 days	Wnt3a↑; β-catenin↑; TCF4↑; Cyclin D1↑; p-GSK3β(Ser9)↓; VEGF, bFGF, Ang2, and FLK1↑; effects blocked by DKK1	Activates the canonical Wnt pathway, promotes angiogenesis, and restores cerebral blood flow in the ischemic region	Shi et al. (2022)
Acupuncture/electroacupuncture	Electroacupuncture (Quchi LI11, Zusanli ST36)	Rat MCAO/R model; NPC assessment in the peri-infarct cortex	*In vivo*	LI11 and ST36, needle insertion 2–3 mm; sparse-dense wave at 1/20 Hz for 30 min once daily; started on postoperative day 1 and continued until sacrifice	Wnt1 mRNA/protein↑; β-catenin mRNA/protein↑; GSK3 transcription↓; nestin/GFAP double-positive NPCs↑	Activates Wnt/β-catenin signaling, promotes NPC proliferation in the peri-infarct cortex, alleviates neurological deficits, and reduces infarct volume	Chen et al. (2015)
Acupuncture/electroacupuncture	EA pretreatment (Baihui GV20)	Rat MCAO/R model; Dkk-1 antagonism and LiCl agonist validation	*In vivo*	GV20 pretreatment for 30 min at 2 h before ischemia; 1 mA, 2/15 Hz; MCAO for 2 h followed by 24 h reperfusion; Dkk-1 administered 30 min before MCAO	Hippocampal β-catenin↑; Bcl-2/Bax↑; Dkk-1 attenuates protection; LiCl increases β-catenin and Bcl-2/Bax	Involves Wnt/β-catenin signaling in EA pretreatment-induced rapid ischemic tolerance; inhibits apoptosis, protects hippocampal CA1 neurons, and improves learning and memory	He et al. (2016)
Acupuncture/electroacupuncture	EA Xingnao Kaiqiao acupoint prescription	Permanent MCAO model	*In vivo*	Not stated in the source text	VEGF↑; GFAP↑; NeuN↑; β-catenin↑; Axin2↓	Activates canonical Wnt/β-catenin signaling and repairs the damaged neurovascular unit	Li et al. (2021)
Acupuncture/electroacupuncture	EA mediated by exosomal miR-210	C57BL/6J mouse MCAO model; SH-SY5Y cell HIF1A overexpression/knockdown validation	*In vivo*/*in vitro*	Paired head acupoints; sparse-dense wave at 2/100 Hz, 1 mA, 30 min/session; started 12 h after recovery from anesthesia and repeated every 24 h for 21 days	Exosomal miR-210↑; miR-210 targets APC; WNT1↑; HIF1A↑; NTN1↑; VEGF/NTN1 transcription↑	Regulates the Wnt/HIF1A/netrin-1 axis via miR-210, promotes angiogenesis and neural reconstruction, and improves motor function after MCAO	Liang et al. (2025)
Acupuncture/electroacupuncture	Electroacupuncture (Shuigou GV26)	Rat MCAO model	*In vivo*	EA immediately after modeling; detailed course not stated in the source text	miR-34↓; WNT1↑; β-catenin↑; LC3II↓; p62↑	Inhibits excessive autophagy through the miR-34/Wnt/autophagy axis and attenuates ischemic brain injury	Cao et al. (2021)
Acupuncture/electroacupuncture	Electroacupuncture (Shuigou GV26)	Rat permanent MCAO model	*In vivo*	Continuous intervention at multiple time points for 3, 7, and 12 days	Wnt7a↑; LEF1↑; GSK-3β↓; DKK1↓	Maintains sustained Wnt/β-catenin activation, promotes neurogenesis and synaptic remodeling, and improves neurological deficits	Zhang et al. (2020)

CIRI, cerebral ischemia/reperfusion injury; BBB, blood-brain barrier; MCAO/R, middle cerebral artery occlusion/reperfusion; OGD/R, oxygen-glucose deprivation/reoxygenation; BMECs, brain microvascular endothelial cells; EA, electroacupuncture; SVZ, subventricular zone; tPA, tissue plasminogen activator. In the table, “↑” denotes upregulation or activation, whereas “↓” denotes downregulation or inhibition.

### 4.1 Herbal-derived active compounds

#### 4.1.1 Phenolic acids and phenylpropanoids

Phenolic acid and phenylpropanoid compounds appear to modulate CIRI partly through Wnt/β-catenin signaling, mainly by promoting neural repair, improving neurological function, and reducing brain injury.

Salvianolic acid A (SAA), a water-soluble bioactive compound isolated from Salvia miltiorrhiza, has been shown to exert pharmacological effects related to cardiovascular and cerebrovascular protection. Zhang et al. used a rat ischemic stroke model induced by electrocoagulation-generated autologous thrombus to investigate the effects and molecular mechanisms of long-term SAA treatment on neurological deficits after ischemic stroke. *In vivo*, SAA administered by gavage at 10 mg/kg for 14 consecutive days reduced cerebral infarct volume, alleviated vascular embolism, attenuated pathological injury in the hippocampus and striatum, increased survival, and improved motor, sensory, and spontaneous activity-related neurological functions. *In vitro* and tissue-level analyses showed that SAA promoted the proliferation of neural stem/progenitor cells in the subventricular zone, their migration toward the ischemic striatum, and their differentiation into mature neurons. It also enhanced axonal regeneration and reduced neuronal apoptosis. Mechanistically, SAA upregulated Wnt3a, p-GSK-3β, β-catenin, and downstream pathway proteins, including TCF-4, Cyclin D1, NeuroD1, and BDNF. It also increased the Bcl-2/Bax ratio and downregulated cleaved caspase-3/9. These findings suggest that SAA may promote endogenous neurogenesis and inhibit neuronal apoptosis by activating Wnt3a/GSK-3β/β-catenin signaling, providing experimental evidence for neural repair therapy during stroke recovery. However, this study used only a single rat thrombotic stroke model, did not compare different treatment durations or long-term dose gradients, lacked exploration of combined intervention with clinical thrombolytic agents, and did not assess dynamic drug distribution in brain tissue or long-term toxicological safety. In addition, the regulatory effects on hippocampal dentate gyrus neurogenesis were not fully validated, and the interaction between neurogenesis and angiogenesis, as well as the precise upstream and downstream targets of the pathway, requires further confirmation (Zhang et al., 2022).

p-Hydroxybenzaldehyde (p-HBA), a phenolic bioactive compound isolated from Gastrodia elata, can improve the peri-infarct cortical microenvironment after cerebral ischemia/reperfusion. Yuan et al. established a rat transient middle cerebral artery occlusion (tMCAO) model to clarify the effects and molecular mechanisms of p-HBA in brain repair. Previous *in vitro* work showed that p-HBA could induce the transdifferentiation of reactive astrocytes into neurons. *In vivo* results further demonstrated that administration during the acute phase of stroke improved motor deficits in a time-dependent manner. p-HBA alone reduced excessive astrocyte activation, promoted the conversion of peri-infarct cortical reactive astrocytes into neurons, induced angiogenesis, and preserved neurovascular unit integrity. Lineage-tracing experiments confirmed that the newly generated neurons originated from local *in situ* astrocytes rather than migrated neural precursor cells from the subventricular zone. This effect depended on activation of the canonical Wnt/β-catenin pathway, with increased expression of Wnt3a, p-GSK-3β, and β-catenin, thereby initiating neurogenesis and coordinating neurogenesis with angiogenesis to remodel the reparative microenvironment. Notably, p-HBA moderately attenuated, rather than completely eliminated, glial scarring, preserving the protective injury-isolating role of glial scars during the subacute phase. Compared with the positive control drug atorvastatin, p-HBA showed stronger neurorestorative effects. Nevertheless, several limitations remain, including the lack of validation in large animals and humans, unclear phosphorylation sites of GSK-3β and downstream target genes regulated by p-HBA, insufficient characterization of excitatory/inhibitory subtypes and cortical laminar distribution of newly generated neurons, and the need for further optimization of dose, therapeutic window, and long-term neurobehavioral and cognitive safety evaluation (Yuan et al., 2022).

Liu et al. reported that phenylethanoid glycosides from Cistanches Herba (PhGs) promote neural stem cell proliferation through Wnt/β-catenin signaling and thereby improve ischemic brain injury. *In vitro*, treatment with PhGs at 15 or 30 μg/mL significantly increased the viability of subventricular zone neural stem cells subjected to oxygen-glucose deprivation/reoxygenation (OGD/R), increased the proportion of EdU-positive proliferating cells, and reversed ischemia/hypoxia-induced suppression of proliferation. In an *in vivo* mouse MCAO model, gavage administration of PhGs at 100 or 200 mg/kg for seven consecutive days after surgery significantly reduced neurological deficit scores, improved gait and motor function, decreased cerebral infarct volume, and alleviated histopathological brain injury. PhGs also increased the number of Ki67-positive proliferating neural stem cells in the subventricular zone, without causing obvious abnormal loss of animals during administration. Mechanistically, PhGs upregulated Wnt3a, total β-catenin, and nuclear β-catenin protein expression, downregulated the negative regulator GSK-3β, and promoted expression of the downstream target gene c-Myc, thereby activating the canonical Wnt pathway to drive neural stem cell proliferation. The Wnt pathway inhibitor IWR-1 abolished the effects of PhGs on neural stem cell proliferation and brain injury repair, suggesting that PhGs may activate endogenous neurogenesis in a Wnt/β-catenin-dependent manner and alleviate post-ischemic brain dysfunction. However, this study evaluated only the efficacy of 7-day acute-phase treatment, did not assess the persistence of medium- or long-term neural repair, and was limited to cellular and mouse models without validation in large animals or clinical samples. The upstream molecular details by which PhGs regulate GSK-3β activity and β-catenin nuclear transport also remain to be clarified (Liu et al., 2023).

Collectively, these studies suggest that phenolic acid and phenylpropanoid compounds may improve CIRI outcomes mainly by regulating Wnt/β-catenin-related signaling and promoting post-ischemic neural repair.

#### 4.1.2 Flavonoids

Flavonoid compounds may ameliorate CIRI mainly through BBB protection, promotion of neurorepair, and attenuation of brain injury, with mechanisms frequently associated with regulation of Wnt/β-catenin-related signaling.

Galangin, a natural flavonoid isolated from the rhizome of Alpinia officinarum Hance, has been reported to protect the neurovascular unit after focal cerebral ischemia. In an MCAO rat model, galangin improved neurological scores, reduced cerebral infarct volume, attenuated cerebral edema, and decreased Evans blue leakage, indicating preservation of BBB integrity and the neurovascular unit (NVU) microenvironment. Mechanistically, galangin modulated the Wnt/β-catenin pathway coupled with HIF-1α/VEGF signaling, with β-catenin and VEGF proposed as key nodes linking neurovascular protection, angiogenesis, and tissue repair after ischemic injury (Wu et al., 2015). However, several limitations remain: the upstream targets through which galangin regulates the Wnt/β-catenin-HIF-1α/VEGF signaling network were not fully resolved; the causal role of β-catenin or VEGF was not verified using specific pathway inhibitors or genetic interventions; the study was conducted in a focal cerebral ischemia model rather than a typical ischemia/reperfusion paradigm; and evidence from aged animals, animals with vascular risk factors, or comorbid disease models is still lacking. The clinical translational feasibility of galangin-mediated neurovascular unit protection therefore requires further investigation.

Naringenin (NAR), a flavanone enriched in citrus fruits and tomatoes, was investigated in a mouse MCAO/R model to clarify its protective effect against post-ischemic BBB injury and the involvement of GSK-3β/β-catenin signaling. *In vivo*, NAR at 5, 10, and 20 mg/kg reduced neurological deficits, infarct area, and neuronal apoptosis in the ischemic penumbra in a dose-related manner. The 10 mg/kg dose appeared to be optimal, whereas 20 mg/kg provided no obvious additional benefit. At this dose, NAR reduced Evans blue extravasation, upregulated the tight junction proteins ZO-1, Occludin, and Claudin-5, inhibited IL-1β, IL-6, and TNF-α release, and increased endothelial inactive p-GSK-3β (Ser9) and β-catenin levels (Yang et al., 2024). However, this study was limited by the use of a single mouse model, intraperitoneal administration, short-term observation within 7 days after reperfusion, and the lack of long-term neurological follow-up, pharmacokinetic and toxicity assessment, delivery optimization for poor solubility and bioavailability, and endothelial cell-specific genetic validation.

Curcumin, a natural polyphenolic compound isolated from turmeric, has been reported to exert neuroprotective and pro-neurogenic effects. Yang et al. used a mouse global cerebral ischemia model induced by bilateral common carotid artery occlusion to investigate whether curcumin improves post-ischemic cognitive impairment and promotes hippocampal dentate gyrus neurogenesis (Yang X. et al., 2021). Curcumin at 50 and 100 mg/kg/day dose-dependently enhanced hippocampal neural stem cell proliferation and promoted the differentiation and maturation of newborn cells into mature neurons. It also upregulated neurogenic transcription factors, including Ngn2, Pax6, and NeuroD1, increased Wnt3a and β-catenin expression and the p-GSK-3β/GSK-3β ratio, attenuated neuronal necrotic injury in the hippocampal CA1 region, and improved spatial learning and memory deficits. Pretreatment with the Wnt receptor antagonist DKK1 abolished these neurorestorative effects, suggesting that activation of Wnt/β-catenin signaling may be a major mechanism by which Curcumin promotes endogenous neurogenesis (Yang X. et al., 2021). Nevertheless, the study was restricted to a single global ischemia model and lacked validation in focal or aged ischemic models, data on BBB permeability and *in vivo* pharmacokinetics, long-term toxicity evaluation, combination studies with neuroprotective drugs, and direct causal validation of downstream Wnt-regulated neurogenic targets.

Medicarpin, a pterocarpan phytoalexin isolated from Radix Hedysari, has anti-inflammatory, anti-apoptotic, and neurorestorative activities. In a mouse cerebral ischemia/reperfusion model, as well as BV2 microglial and N2A neuronal cell models, Medicarpin inhibited LPS-induced NO production in microglia and alleviated OGD-induced neuronal apoptosis. *In vivo*, Medicarpin at 0.5 and 1.0 mg/kg dose-dependently improved survival, ameliorated sensorimotor deficits, reduced infarct volume, and protected the BBB. It also downregulated p65 NF-κB and activated caspase-3, upregulated DCX, BDNF, TrkB, and Bcl-2, and promoted GSK-3 inactivation and β-catenin accumulation. The PI3K inhibitor LY294002 abolished these protective effects, suggesting that Medicarpin may activate PI3K/Akt signaling to inhibit GSK-3, stabilize β-catenin, suppress neuroinflammation and neuronal apoptosis, and induce endogenous neurogenesis. These findings support its potential as a natural bioactive candidate for ischemic stroke (Chern et al., 2021). However, the evidence remains limited by the use of a single ICR mouse model, insufficient assessment across animal strains, lack of long-term safety and pharmacokinetic evaluation, absence of combination studies with rt-PA, and incomplete genetic validation of the causal relationship between cellular effects and target regulation in brain tissue.

Overall, flavonoid compounds may exert neuroprotective effects at multiple levels, including BBB preservation, neurogenesis promotion, and reduction of brain injury, partly through modulation of Wnt/β-catenin-related signaling.

#### 4.1.3 Terpenoids and lactones

Terpenoid and lactone monomers mainly intervene in CIRI by promoting neural regeneration and astrocyte-to-neuron transdifferentiation, and their effects may be associated with regulation of Wnt/β-catenin-related signaling.

To clarify the neuroprotective effects of L-borneolum and D-borneolum during the recovery phase of cerebral ischemia, as well as their distinct molecular mechanisms, Ma Rong et al. established a rat transient middle cerebral artery occlusion (tMCAO) ischemia/reperfusion model. *In vivo* pharmacodynamic results showed that continuous intragastric administration of either borneol enantiomer at 50–200 mg/kg significantly restored neuromotor function, increased cerebral blood flow in the ischemic region, and reduced cerebral infarction and brain atrophy, without causing liver, kidney, or gastric mucosal injury during the treatment period. L-borneolum showed a more prominent advantage in improving limb coordination, downregulated Wnt3a and Notch-1, and promoted the conversion of striatal astrocytes into mature gamma-aminobutyric acid (GABA)-ergic neurons. By contrast, D-borneolum showed stronger effects in improving limb tactile function and inhibiting brain atrophy, upregulated GSK-3β/β-catenin signaling, and induced astrocyte conversion into nestin-positive neural stem cells. The effects of both enantiomers were independent of vascular endothelial growth factor (VEGF), were restricted to the striatum and subventricular zone, and showed no obvious neural repair effects in the cortex or hippocampus. This study confirmed that the two compounds exert neuroprotection by regulating the Wnt/Notch pathway, remodeling cerebral microcirculation, and mediating astrocyte-to-neuron transdifferentiation. Overall, the two chiral borneol compounds exert neuroprotective effects during stroke recovery through differential pathway regulation, improvement of the cerebral microenvironment, and promotion of glial-cell transdifferentiation, suggesting their potential value as candidate natural products for stroke rehabilitation (Ma et al., 2023). However, this study has clear limitations: glial transdifferentiation was judged only by fluorescence tracing, without reverse validation using gene knockout; the electrophysiological function of newly generated neurons was not assessed; the action targets corresponding to differences in chiral structure were not resolved; and safety was evaluated only in the short term, with no long-term toxicity data supporting repeated administration.

Wang et al. revealed that senkyunolide I (SEI) promotes neural stem/progenitor cell proliferation through the Akt/β-catenin pathway. *In vitro*, neural stem/progenitor cells derived from embryonic rats were treated with gradient concentrations of SEI, and SEI at 200 μM and 400 μM increased cell proliferative activity in a dose-dependent manner. Mechanistically, SEI upregulated Akt phosphorylation and increased active β-catenin expression, while the phosphoinositide 3-kinase (PI3K) inhibitor LY294002 completely reversed the pro-proliferative effect of SEI, confirming that this effect depends on activation of the PI3K/Akt pathway. This process was not accompanied by changes in GSK-3β phosphorylation at Ser9. The study proposed that SEI may preferentially regulate symmetric division of neural stem/progenitor cells to expand the stem-cell pool, suggesting its potential as a candidate agent for endogenous neural repair in brain injury and neurodegenerative diseases (Wang et al., 2023). However, the study was limited to *in vitro* cellular experiments and did not verify *in vivo* efficacy or drug distribution in brain tissue. It also lacked clarification of the complete upstream molecular cascade by which SEI regulates active β-catenin, did not distinguish the differential regulation of β-catenin by the canonical Wnt pathway and the Akt bypass pathway, and did not compare different primary neural cells or stem-cell subtypes. Because the conclusions were supported by only a single embryonic rat cell model, key translational data, including long-term effects across the BBB and safe *in vivo* dosing, remain lacking. Further validation through *in vivo* animal experiments, multi-level mechanistic studies, and multi-species cellular models is still required.

In summary, terpenoid and lactone monomers may participate in post-ischemic neural repair by regulating β-catenin-related signaling.

#### 4.1.4 Saponins

Saponin compounds mainly intervene in CIRI through inhibition of neuroinflammation, promotion of hippocampal neurogenesis, and post-ischemic white matter repair, and these effects are largely associated with regulation of Wnt/β-catenin-related signaling.

Ilexonin A (IA), a pentacyclic triterpene extracted from the Chinese medicine *Ilex pubescens*, has also been shown to promote neuronal proliferation and regeneration after cerebral ischemia/reperfusion. In a rat MCAO/R model, IA improved neurological deficits and increased the numbers of BrdU/nestin and BrdU/NeuN double-positive cells in peri-ischemic tissue. Mechanistically, IA increased β-catenin protein and LEF1 mRNA levels while decreasing GSK-3β protein and Axin mRNA expression, suggesting activation of the canonical Wnt pathway and enhancement of post-ischemic neuroregeneration (Zhang et al., 2016). These findings support IA as a potential pro-regenerative compound acting on the GSK-3β/β-catenin/LEF1 axis. Nevertheless, the evidence remains mainly correlative, because pathway activation was inferred from changes in signaling molecules rather than validated through pathway blockade or genetic manipulation. Further work is therefore needed to define the direct molecular targets of IA and to determine whether its regenerative effects persist in aged animals or models with vascular comorbidities.

Ginsenoside Rb1 is a highly active natural protopanaxadiol-type ginsenoside derived from Panax ginseng. It has anti-inflammatory, antioxidant, and anti-apoptotic pharmacological activities and shows potential for intervention in central nervous system injury. Liu et al. investigated the neuroprotective effects and molecular mechanisms of Ginsenoside Rb1 using an *in vitro* OGD/R model in BV2 microglia and an *in vivo* mouse MCAO/R model of CIRI. Their results showed that Ginsenoside Rb1 alleviated CIRI by regulating microglial polarization and activating the Wnt/β-catenin pathway. *In vitro*, Ginsenoside Rb1 induced hypoxia-stressed microglia to shift from the pro-inflammatory M1 phenotype toward the anti-inflammatory M2 phenotype, downregulated the pro-inflammatory factors TNF-α and IL-1β, and upregulated the anti-inflammatory markers Arg-1 and CD206. Mechanistically, Ginsenoside Rb1 inhibited GSK-3β expression and increased β-catenin protein levels, thereby activating Wnt signaling; the pathway inhibitor XAV939 completely blocked its anti-inflammatory and protective effects. In the mouse model, Ginsenoside Rb1 alleviated neurological deficits, reduced cerebral infarct area, restored cerebral blood flow, and promoted VEGF-mediated angiogenesis, whereas co-treatment with XAV939 abolished all pharmacological effects. No organ injury was observed after administration. This study provides a potential new target for active TCM components in ischemic stroke intervention. However, several limitations remain: the upstream molecules through which Ginsenoside Rb1 regulates GSK-3β/β-catenin were not resolved; target validation relied only on a pathway inhibitor; no combination comparison with clinically used thrombolytic agents was performed; only healthy young mice were used; and evidence from aged animals or animals with underlying diseases is lacking. The clinical translational feasibility of these findings therefore requires further investigation (Liu R.-J. et al., 2025).

Astragaloside IV (As IV) is a core active compound isolated from Astragali Radix (the dried root of *Astragalus membranaceus*) and has been shown to possess anti-inflammatory, neuroprotective, and tissue-repair activities. Sun et al. established a photochemically induced mouse model of ischemic stroke and combined it with an *in vitro* culture system of embryonic hippocampal neural stem cells to investigate the effects and molecular mechanisms of As IV in improving post-stroke cognitive deficits and promoting hippocampal neurogenesis. *In vivo*, intravenous administration of As IV at 2 mg/kg significantly restored spatial learning and memory, improved dendritic structure in hippocampal neurons, increased dendritic spine density, and upregulated DCX, BrdU, Sox2, and other markers to promote neural stem cell proliferation. *In vitro*, As IV at 10 and 100 nM increased neurosphere size and maintained stemness. Both *in vivo* and *in vitro* experiments showed that As IV significantly downregulated the stroke-induced high expression of IL-17, and IL-17 knockout mice reproduced its neurorepair effects. The core mechanism involved inhibition of IL-17, followed by upregulation of Wnt2 and β-catenin and downregulation of GSK-3β, resulting in activation of the Wnt pathway. Transcriptome sequencing further supported that IL-17 deficiency could reverse ischemia-induced disturbances in synaptic and pathway-related genes, providing experimental evidence for intervention in ischemic stroke-related cognitive impairment (Sun et al., 2020). Nevertheless, this study used only a single photothrombotic ischemia model and lacked comparison with an ischemia/reperfusion model. Pharmacokinetic analysis and BBB penetration assessment were not performed. IL-17 overexpression rescue experiments were lacking, limiting direct validation of the upstream-downstream relationship of the pathway. In addition, no combination therapy or long-term safety evaluation was conducted, and the relevance of this target to brain tissue from clinical patients remains to be further verified.

TSTT is a total saponins fraction extracted and isolated from the rhizomes of *Trillium tschonoskii* Maxim. and has been shown to possess neuroprotective and neural repair-promoting activities. Yang et al. used a rat model of permanent middle cerebral artery occlusion to investigate the effects and molecular mechanisms of TSTT in promoting post-stroke oligodendrogenesis and axonal remodeling. *In vivo*, TSTT at 33 and 65 mg/kg significantly improved motor deficit scores and beam-walking performance in rats. Multiparametric magnetic resonance imaging showed that TSTT reduced cerebral infarct volume, increased fractional anisotropy, fiber length, and fiber density in the sensorimotor cortex and internal capsule, decreased transverse relaxation time and radial diffusivity, and alleviated demyelination and axonal ultrastructural injury. Histological results showed that TSTT significantly increased the numbers of Ki67/NG2 and Ki67/CNPase double-positive cells around the infarct, promoted proliferation and differentiation of oligodendrocyte precursor cells, upregulated phosphorylation of GSK-3β at Ser9, and downregulated phosphorylated β-catenin and CRMP-2 expression. These effects regulated key signaling pathways involved in axonal remodeling and ultimately promoted remyelination, axonal reconstruction, and motor functional recovery. The core mechanism was attributed to regulation of the GSK-3/β-catenin/CRMP-2 signaling axis and activation of endogenous oligodendrogenesis, providing experimental support for white matter injury repair after ischemic stroke (Yang L. et al., 2021). However, this study used only male rats in a single ischemia model and did not include female animals to evaluate sex differences. Dynamic observations across different time gradients were lacking. The *in vivo* absorption, metabolism, and BBB penetration of TSTT were not investigated. Validation of a single pathway is insufficient to fully explain the neural repair effects of multi-target saponins, and the independent and synergistic effects of individual active monomers, as well as the safety of long-term administration, remain to be further examined.

In summary, saponin compounds mainly exert neuroprotective effects by regulating Wnt/β-catenin-related molecular nodes and acting on inflammatory responses, neurogenesis, and white matter repair.

#### 4.1.5 Other compounds

Mallotus oblongifolius (MO), an edible medicinal plant traditionally consumed as herbal tea in southern China, has been reported to ameliorate ischemic nerve injury through activation of Wnt/β-catenin signaling. In a mouse model of cerebral ischemia/reperfusion induced by bilateral common carotid artery ligation, MO water extract improved motor, neurosensory, balance, and grasping abilities, preserved neuronal morphology, and increased nestin expression in the cerebral cortex and subgranular zone. In an OGD/R-treated neural stem cell model, MO extract promoted NSC proliferation and increased the expression of β-catenin and Cyclin D1, suggesting that activation of the Wnt/β-catenin/Cyclin D1 axis may contribute to endogenous neural repair after ischemic injury (Li et al., 2020). This study broadens the evidence for Wnt/β-catenin-mediated neurorepair from purified compounds to whole herbal extracts, suggesting that edible medicinal plants may also promote endogenous NSC proliferation after ischemic injury. Nevertheless, because MO was administered as a crude water extract and the active constituents responsible for Wnt/β-catenin activation were not identified, further work is needed to define its chemical basis, direct molecular targets, and reproducibility in more standardized CIRI models.

Hirudin is a polypeptide active substance extracted from the salivary glands of leeches and has potent anticoagulant activity. Existing evidence suggests that it can promote angiogenesis and exert neuroprotective effects. He et al. used an *in vitro* OGD/R model of brain microvascular endothelial cells and a rat MCAO/R cerebral ischemia-reperfusion model to clarify its protective mechanism in ischemic stroke. In rat brain microvascular endothelial cells (BMECs), hirudin at 20, 40, and 80 μg/mL significantly increased endothelial cell viability after OGD/R injury, enhanced cell migration and tube formation, and improved angiogenic potential. *In vivo*, intraperitoneal administration of hirudin at 40 mg/kg for 7 days reduced neurological deficit scores, decreased cerebral infarct volume, alleviated pathological injury in the ischemic penumbra, and upregulated the expression of the angiogenic markers CD34, VEGF, and Ang-2. Hirudin also increased the protein and mRNA levels of Wnt3a and β-catenin, thereby activating the canonical Wnt/β-catenin pathway. The pathway inhibitor DKK1 reversed its pro-angiogenic effects, indicating that hirudin promotes angiogenesis in the ischemic penumbra and achieves neuroprotection through activation of Wnt/β-catenin signaling. This study comprehensively confirms the stroke-protective effect of hirudin-mediated cerebral angiogenesis (He et al., 2025). However, several limitations remain. The *in vivo* experiments used only a single dose and did not include graded animal dosing; pharmacokinetic analyses linking *in vitro* and *in vivo* drug concentrations were absent, making it difficult to define the effective exposure dose *in vivo*; and the risk of intracranial hemorrhage associated with the strong anticoagulant activity of hirudin was not evaluated. Potential adverse effects, such as abnormal permeability of newly formed vessels, also require systematic validation. In addition, the mechanistic analysis focused on a single pathway and lacked cross-validation of a broader regulatory network.

Echinacoside (ECH) is a naturally derived small-molecule compound with neuroprotective activity. Zeng et al. investigated its anti-ischemic effects and molecular mechanism through multi-level experiments. In an *in vitro* OGD/R cell model, ECH at 2.5–10 μmol/L significantly increased PC12 cell viability, reduced LDH release, alleviated mitochondrial fragmentation, maintained mitochondrial membrane potential and mitochondrial DNA homeostasis, and upregulated the key mitochondrial fusion protein Mfn2. Molecular interaction assays showed that ECH selectively formed a covalent interaction with the K171 site of the CK2α′ subunit, allosterically regulating protein conformation without affecting the classical kinase activity of CK2. This interaction recruited BTF3α to form a binary complex, which activated the Wnt/β-catenin pathway, promoted β-catenin nuclear translocation, and drove Mfn2 transcription to enhance mitochondrial fusion. In a mouse MCAO model, ECH improved brain injury and neurobehavioral deficits in wild-type mice, whereas this protective effect was largely abolished in CK2α′ heterozygous knockout mice. These results indicate that ECH exerts neuroprotective effects against ischemic stroke by targeting CK2α′, activating Wnt/β-catenin signaling, and upregulating Mfn2-mediated mitochondrial fusion (Zeng et al., 2021; Zeng et al., 2025). Nevertheless, this study validated only a single pathway for one natural small molecule and did not examine drug metabolism or BBB penetration. Long-term safety data and large-animal efficacy evidence are also lacking. The potential off-target toxicity associated with covalent CK2α′ binding and the synergistic or antagonistic effects of ECH combined with clinical thrombolytic agents remain unexplored, leaving substantial gaps before clinical translation can be considered.

### 4.2 Chinese herbal compound formulas and related patent preparations

Available evidence suggests that Chinese herbal compound formulas and related patent preparations can intervene in CIRI through regulation of Wnt/β-catenin signaling at multiple levels, including inhibition of neuroinflammation, protection of the BBB, promotion of neuroregeneration, and enhancement of angiogenesis.

Danggui-Jakyak-San (DJS), also known as Danggui-Shaoyao-San in Chinese and Toki-Shakuyaku-San in Japanese, is a traditional herbal prescription composed of Paeoniae Radix, Atractylodis Rhizoma, Alismatis Rhizoma, Hoelen, Cnidii Rhizoma, and Angelicae Gigantis Radix. In a mouse model of transient forebrain ischemia induced by bilateral common carotid artery occlusion, delayed long-term administration of DJS ameliorated ischemia-induced spatial memory impairment and increased hippocampal neurogenesis in the dentate gyrus. Mechanistically, DJS increased the expression of p-Akt, p-GSK-3β, and β-catenin in the hippocampus, suggesting that activation of the Akt/GSK-3β/β-catenin signaling axis may contribute to its pro-neurogenic and cognitive benefits after ischemic injury (Song et al., 2013). Although this model does not fully reproduce focal CIRI, this study provides supportive evidence that classical herbal prescriptions may enhance delayed hippocampal neurogenesis and cognitive repair through the Akt/GSK-3β/β-catenin axis.

Fuzheng Jiedu Tongluo Granule (FZJDTL), a Chinese herbal preparation derived from a classical formula, has been reported to mitigate BBB disruption after ischemic stroke by regulating endothelial transcytosis. In MCAO/R rats and OGD/R-injured bEnd.3 endothelial cells, FZJDTL reduced neurological deficit scores, infarct volume, Evans blue extravasation, brain water content, albumin leakage, and sodium fluorescein permeability, while preserving TEER values and BBB integrity. Mechanistically, MCAO/R or OGD/R decreased Wnt3a, β-catenin, Mfsd2a, and claudin-5 expression and increased Cav-1 expression, indicating suppression of Wnt/β-catenin signaling, loss of the vesicle-inhibitory protein Mfsd2a, enhanced Cav-1-mediated transcytosis, and tight junction impairment. FZJDTL reversed these changes by upregulating Wnt3a, β-catenin, Mfsd2a, and claudin-5 while downregulating Cav-1, thereby suppressing endothelial transcytosis and preserving BBB barrier function (Liu et al., 2026). This study is particularly relevant to BBB-centered CIRI mechanisms because it links Wnt/β-catenin activation with Mfsd2a-mediated inhibition of Cav-1-dependent transcytosis, a process less frequently discussed than tight junction degradation in post-ischemic barrier injury.

Danhong Injection (DHI), a standardized TCM injection composed of extracts from Radix Salvia miltiorrhiza and Carthamus tinctorius L., has been reported to protect BBB integrity under hyperlipidemia-aggravated cerebral ischemia/reperfusion conditions. In hyperlipidemic rats subjected to MCAO/R, DHI improved neurological function, reduced infarct volume, alleviated cortical pathological injury, and preserved BBB ultrastructure. At the molecular level, ischemia/reperfusion combined with hyperlipidemia downregulated the tight junction proteins Claudin-5, Occludin, and ZO-1, increased MMP-9 expression, reduced Wnt3α, p-GSK-3β, Cyclin D1, and nuclear β-catenin levels, and promoted cytoplasmic β-catenin accumulation. DHI reversed these alterations by upregulating Claudin-5, Occludin, ZO-1, Wnt3α, p-GSK-3β, Cyclin D1, and nuclear β-catenin, while downregulating MMP-9 and cytoplasmic β-catenin, thereby activating Wnt/β-catenin signaling and preserving BBB integrity (Shi et al., 2026). The reversal of DHI-mediated protection by DKK1 further supports the involvement of Wnt/β-catenin signaling, especially under hyperlipidemia-aggravated CIRI conditions.

Yangyin Yiqi Huoxue prescription (YYH), composed of Puerariae Lobatae Radix, Astragali Radix, Rehmanniae Radix, Chuanxiong Rhizoma, Hirudo, and Dendrobii Officinalis Caulis, has the effects of nourishing yin, tonifying qi, activating blood circulation, and dredging collaterals. As a new traditional Chinese medicine that has completed phase II and phase III clinical trials, YYH is used for both the acute and recovery phases of ischemic stroke. Lu et al. investigated its reparative effects and molecular mechanisms using a rat MCAO model, BV2 and HT22 cell OGD/R injury models, and a co-culture system. *In vivo*, YYH at 0.83, 1.65, and 3.3 g/kg significantly improved neurological deficits, reduced cerebral infarct volume, decreased the level of the brain injury marker S100β, and alleviated neuronal Nissl body damage. Mechanistically, YYH activated the canonical Wnt pathway by upregulating Wnt3a and β-catenin and downregulating GSK-3β, thereby promoting neural stem cell proliferation and differentiation and inhibiting glial scar formation. It also downregulated Wnt5a to suppress the non-canonical Wnt pathway, inhibited excessive microglial activation, reduced the release of IL-1β, IL-6, and TNF-α, and attenuated neuroinflammation. *In vitro*, 10% YYH-containing serum scavenged microglial ROS, promoted M1-to-M2 polarization, inhibited the NLRP3 inflammasome, and reduced neuronal apoptosis. These effects were reversed by the Wnt pathway inhibitor DKK1, supporting the Wnt pathway as a central mechanism of YYH action (Lu et al., 2025). These findings suggest that YYH may coordinate canonical and non-canonical Wnt signaling to promote neural repair while suppressing microglia-mediated neuroinflammation.

Huang-Lian-Jie-Du Decoction (HLJDT), a classical formula recorded in the Tang-dynasty text Wai Tai Mi Yao, has demonstrated notable neuroprotective and anti-inflammatory activities. Chen et al. used a rat MCAO cerebral ischemia-reperfusion model to examine how HLJDT inhibits central infiltration of CD4^+^ T cell and alleviates post-stroke neuroimmune inflammation. Medium-dose HLJDT at 0.9 g/kg improved neurological deficits, reduced cerebral infarct volume, and attenuated neuronal necrosis and Nissl body loss. HLJDT balanced CD4^+^ T-cell immune phenotypes by downregulating the Th1/Th17 pro-inflammatory factors IFN-γ and IL-17A and upregulating the Th2/Treg anti-inflammatory factors IL-4 and TGF-β. It also blocked LFA-1/ICAM-1-mediated adhesion, repaired both transcellular and paracellular BBB pathways, upregulated Occludin, E-cadherin, and Mfsd2a, downregulated the vesicular transport protein Cav-1, and reduced vascular leakage. The core mechanism was attributed to activation of the Wnt7a/β-catenin pathway, which simultaneously repaired BBB structure and prevented CD4^+^ T-cell infiltration into the central nervous system. These findings provide experimental support for targeting neuroimmune inflammation in ischemic stroke (Chen et al., 2025). These findings suggest that the Wnt7a/β-catenin pathway may link BBB repair with reduced CD4^+^ T-cell infiltration and neuroimmune inflammation after ischemic stroke.

Buyang Huanwu Decoction (BHD), first described by Wang Qingren in Yilin Gaicuo during the Qing dynasty, consists of Astragali Radix, Angelicae Sinensis Radix, Paeoniae Radix Rubra, Pheretima, Chuanxiong Rhizoma, Persicae Semen, and Carthami Flos. It has clear therapeutic relevance for neurological injury and neural regeneration after cerebral ischemia. Ou et al. established a mouse MCAO model and combined it with Cav1 knockout mice to verify the genetic function underlying BHD-mediated neurogenesis. *In vivo*, 18.5 g/kg BHD was identified as the optimal effective dose, significantly improving neurobehavioral deficits, reducing cerebral infarct volume, and alleviating cortical neuronal injury. Mechanistically, BHD upregulated the membrane structural protein Cav1, activated the canonical Wnt/β-catenin pathway, increased Wnt1 and β-catenin expression, and downregulated GSK3β. These effects promoted hippocampal neural stem cell proliferation, differentiation and maturation of newborn neurons, and secretion of neurotrophic factors such as BDNF, NGF, and GDNF, thereby repairing ischemic brain tissue and enhancing endogenous neural regeneration. Cav1 knockout markedly weakened the neuroprotective and pathway-activating effects of BHD, confirming Cav1 as a key upstream target (OuYang et al., 2025). The use of Cav1 knockout mice strengthens the evidence that Cav1 may function as an upstream regulator of BHD-induced Wnt/β-catenin activation and endogenous neurogenesis.

Gujin Luyan Xuming Decoction (XMD), a classical formula recorded in Jin Gui Yao Lue for the treatment of cerebral infarction, contains multiple herbs, including Ephedrae Herba, Cinnamomi Ramulus, and Chuanxiong Rhizoma. One study used a rat MCAO/R model and a bEnd.3 endothelial cell OGD/R model to evaluate its effects. Rats received XMD by gavage at 10.26, 20.52, or 41.04 g/kg/day, while cells were treated with 10% drug-containing serum. High-dose XMD dose-dependently reduced infarct volume, restored neurological function, improved pathological brain injury, enhanced the viability of injured endothelial cells, and promoted cell migration and tube formation. LC-MS/MS identified 33 blood-entering active components. By integrating transcriptomics, single-cell sequencing, and network pharmacology, RAB11A was identified as a core target, and molecular docking and molecular dynamics simulations suggested stable binding between active components and RAB11A. Both *in vivo* and *in vitro* experiments showed that XMD upregulated RAB11A, Wnt5a, β-catenin, VEGFA, and VEGFR2, indicating that it promotes angiogenesis and alleviates cerebral ischemic injury by activating the RAB11A-mediated Wnt5a/β-catenin pathway. This multi-omics study provides mechanistic evidence for the angiogenic effects of XMD and supports the use of traditional Chinese medicine in stroke treatment (Cai et al., 2026). This multi-omics study suggests that XMD may promote post-ischemic angiogenesis through the RAB11A-mediated Wnt5a/β-catenin/VEGFA-VEGFR2 axis.

Dandeng Tongnao Capsules are a Chinese patent medicine used clinically for ischemic stroke and consist of Salviae Miltiorrhizae Radix et Rhizoma, Erigerontis Herba, Chuanxiong Rhizoma, and Puerariae Lobatae Radix. A dual-model study using rat MCAO and OGD/R-injured brain microvascular endothelial cells (BMECs) found that Dandeng Tongnao Capsule-containing cerebrospinal fluid at concentrations of 1%–20% significantly reduced cerebral infarct volume, improved neurological deficits, and increased cerebral microvessel density in rats. *In vitro*, the medicated cerebrospinal fluid increased the survival of OGD/R-injured BMECs, downregulated Bax, and upregulated Bcl-2. Mechanistic analysis showed that Dandeng Tongnao Capsules upregulated Wnt-1, β-catenin, and LRP-6 and downregulated GSK-3β, thereby activating the Wnt/β-catenin signaling pathway, attenuating cerebral microvascular endothelial injury, protecting the BBB, and exerting anti-ischemic effects. By combining cerebrospinal fluid pharmacology with *in vivo* and *in vitro* models, this study provides modern experimental evidence for the pharmacodynamic basis and signaling mechanism of Dandeng Tongnao Capsules in ischemic stroke (Huang et al., 2022). This study provides evidence that Dandeng Tongnao Capsules may protect cerebral microvascular endothelial cells and the BBB through activation of the Wnt-1/LRP-6/β-catenin pathway.

Overall, these studies indicate that Chinese herbal compound formulas and related patent preparations may regulate Wnt/β-catenin signaling as a convergent mechanism linking BBB protection, neuroimmune modulation, angiogenesis, and endogenous neural repair after ischemic injury. However, the current evidence remains limited by heterogeneous ischemia models, insufficient identification of active constituents, incomplete pharmacokinetic and brain-exposure data, and uneven pathway-specific validation. In addition, most studies were performed in rodent models, with limited evidence from aged animals, female animals, comorbidity models, large animals, or clinical samples. These gaps should be addressed before the Wnt/β-catenin-mediated effects of formula-based interventions can be translated into optimized clinical strategies for CIRI.

## 5 Acupuncture therapies in CIRI

Current evidence suggests that acupuncture may intervene in cerebral ischemia-reperfusion injury (CIRI) through regulation of the Wnt/β-catenin signaling pathway. Its effects are mainly reflected in promoting post-ischemic vascular reconstruction, protecting the neurovascular unit, and modulating upstream molecular networks.

Shi et al. showed that scalp electroacupuncture at Baihui (GV20) promotes angiogenesis after cerebral ischemia-reperfusion injury in rats by activating the Wnt/β-catenin pathway. In their animal experiment, a rat model of middle cerebral artery occlusion/reperfusion (MCAO/R) was established, and scalp electroacupuncture at Baihui was administered for 30 min daily for 7, 14, or 21 days. Compared with the model group, the electroacupuncture group showed significantly reduced modified neurological severity scores, smaller cerebral infarct areas, and restored local cerebral blood flow in the ischemic cortex. H&E staining further showed attenuated pathological brain injury and increased numbers of neuronal cells. Protein and mRNA analyses of brain tissue demonstrated synchronous upregulation of pro-angiogenic factors, including VEGF, bFGF, Ang2, and FLK1, while immunohistochemistry confirmed increased expression of angiogenesis-related markers. Mechanistically, scalp electroacupuncture upregulated Wnt3a, β-catenin, TCF4, and Cyclin D1 and downregulated p-GSK3β (Ser9), indicating activation of the canonical Wnt pathway and induction of downstream angiogenic gene transcription to restore blood supply in the ischemic region. Pretreatment with the pathway-specific inhibitor DKK1 abolished the neuroprotective and pro-angiogenic effects of electroacupuncture, confirming that its therapeutic efficacy depends on activation of this pathway (Shi et al., 2022). However, this study was limited to *in vivo* experiments in rats and lacked *ex vivo* or cellular evidence to support a direct regulatory relationship. It also did not include comparisons of different acupoints or electroacupuncture parameters, making it difficult to confirm the specificity of Baihui stimulation. In addition, long-term brain repair outcomes and human clinical data were absent, and the interactive mechanisms by which Wnt signaling regulates angiogenesis were not fully elucidated. Further cellular experiments, multi-control designs, and clinical cohort studies are needed to strengthen these conclusions.

Electroacupuncture (EA) has also been shown to promote endogenous neural repair after cerebral ischemia/reperfusion by activating Wnt/β-catenin signaling. In a rat MCAO/R model, EA stimulation at Quchi (LI11) and Zusanli (ST36) significantly improved neurological deficits, reduced cerebral infarct volume, and increased the number of nestin/GFAP-positive neural progenitor cells in the cortical peri-infarct area. Mechanistically, cerebral I/R reduced Wnt1 and β-catenin expression while increasing GSK3 transcription in the ischemic cortex, whereas EA reversed these changes by upregulating Wnt1 and β-catenin and suppressing GSK3 expression, suggesting activation of the Wnt/β-catenin pathway and enhancement of neural progenitor cell proliferation after ischemic injury (Chen et al., 2015). This study provides direct evidence that EA may contribute to post-ischemic cortical repair by enhancing the proliferative response of endogenous neural progenitor cells in the peri-infarct region. Compared with pharmacological interventions, EA may act through activity-dependent modulation of repair-related signaling networks; however, the causal contribution of Wnt/β-catenin signaling was mainly inferred from expression changes, and further studies using pathway blockade or genetic approaches are needed to verify whether this pathway is indispensable for EA-induced neurogenesis.

He et al. further explored the role of Wnt/β-catenin signaling in electroacupuncture pretreatment-induced ischemic tolerance. In a rat MCAO/R model, electroacupuncture pretreatment at Baihui (GV20) for 30 min, performed 2 h before ischemia induction, increased β-catenin expression in hippocampal neurons 24 h after reperfusion. Functionally, electroacupuncture pretreatment improved Garcia neurological scores, reduced neuronal loss in the hippocampal CA1 region, inhibited apoptosis, and alleviated learning and memory deficits after reperfusion. Mechanistically, these protective effects were associated with upregulation of β-catenin and an increased Bcl-2/Bax ratio, whereas administration of the Wnt/β-catenin antagonist Dkk-1 suppressed β-catenin expression and attenuated the neuroprotective effects of electroacupuncture. Conversely, the Wnt/β-catenin agonist lithium chloride increased β-catenin expression and the Bcl-2/Bax ratio, further supporting the involvement of Wnt/β-catenin signaling in electroacupuncture-induced ischemic tolerance (He et al., 2016). Subsequent studies broadened the focus from neuronal survival and progenitor proliferation to neurovascular remodeling. Li et al. showed that EA based on the Xingnao Kaiqiao acupoint prescription increased VEGF, GFAP, NeuN, and β-catenin expression while downregulating Axin2 after permanent MCAO, indicating activation of canonical Wnt/β-catenin signaling and repair of the damaged neurovascular unit (Li et al., 2021). More recently, Liang et al. proposed a more upstream regulatory mechanism, showing that EA increased exosomal miR-210 and upregulated WNT1, HIF1A, and NTN1 expression. Mechanistically, miR-210 targeted APC, a negative regulator of Wnt signaling, while HIF1A activated VEGF and NTN1 transcription, thereby linking Wnt activation with angiogenesis, axon guidance, and neural reconstruction (Liang et al., 2025). Taken together, these studies suggest that EA may regulate Wnt-related signaling at multiple stages of ischemic brain repair, including anti-apoptotic protection, endogenous progenitor cell proliferation, neurovascular unit remodeling, angiogenesis, and axonal reconstruction. Nevertheless, the current evidence remains heterogeneous. The included studies used different ischemia models, intervention windows, acupoint prescriptions, and stimulation parameters, ranging from EA pretreatment to post-ischemic rehabilitation. Except for partial pathway validation using Dkk-1 or lithium chloride, most studies inferred Wnt/β-catenin involvement mainly from changes in pathway-associated proteins rather than from targeted loss-of-function experiments. In addition, most data were obtained from rodent models, and evidence from female animals, aged animals, comorbidity models, large animals, and clinical samples remains limited. Therefore, the causal role of Wnt/β-catenin signaling in EA-mediated repair and the optimal clinically translatable EA protocol still require systematic validation.

Cao et al. established a rat middle cerebral artery occlusion (MCAO) model and used electroacupuncture at Shuigou (GV26) to systematically investigate its effects and molecular mechanisms in ischemic brain injury. Histological analyses showed that MCAO markedly increased cerebral infarct volume and induced extensive neuronal apoptosis, whereas electroacupuncture significantly reduced infarct size, improved pathological brain injury, and inhibited neuronal apoptosis. High-throughput sequencing identified miR-34, miR-235, and miR-275 as significantly differentially expressed between groups. Among them, miR-34 was markedly upregulated in the cerebral ischemia model, and electroacupuncture reversed this increase. KEGG enrichment analysis suggested that the Wnt signaling pathway was a core regulatory pathway, and dual-luciferase reporter assays confirmed WNT1 as a direct target gene of miR-34. Electroacupuncture inhibited miR-34, thereby releasing WNT1, upregulating β-catenin, and activating the Wnt pathway. This process downregulated LC3II and upregulated p62, suppressing excessive autophagy and ultimately alleviating ischemic brain injury through the miR-34/Wnt/autophagy axis. This study identified a non-coding RNA-mediated regulatory pathway for electroacupuncture treatment of ischemic stroke and provided molecular experimental support for mechanistic research on acupuncture in brain disorders (Cao et al., 2021). However, several limitations remain. Electroacupuncture was applied only immediately after modeling, and the study did not distinguish therapeutic windows in the acute and recovery phases. It lacked reverse validation using a Wnt pathway-specific inhibitor, did not include behavioral neurological scoring to support functional recovery, and did not explore the upstream and downstream regulatory network of miR-34 or the effects of different acupoints and stimulation parameters on this pathway. Therefore, the animal findings cannot be directly translated to clinical practice. Future studies should incorporate behavioral assessment, pathway blockade and rescue experiments, and multi-gradient comparisons of electroacupuncture parameters to complete the evidence chain.

Zhang used a permanent MCAO rat model to reveal the Wnt signaling-related mechanism by which electroacupuncture improves neurological deficits after cerebral infarction. Molecular analyses showed that Wnt pathway-related molecules were spontaneously activated in brain tissue after MCAO modeling, and electroacupuncture further upregulated Wnt7a and LEF1 at both mRNA and protein levels. At the same time, it significantly downregulated the nucleic acid and protein levels of the pathway inhibitors GSK-3β and DKK1, thereby advancing and amplifying pathway activation. Electroacupuncture also reduced neurological severity scores at 3, 7, and 12 days, alleviating motor, sensory, and balance-related neurological deficits. Mechanistically, electroacupuncture maintained sustained activation of the Wnt/β-catenin pathway through dual actions: inhibition of GSK-3β degradation-complex activity and blockade of the receptor-antagonistic effect of DKK1. These effects may promote neurogenesis and synaptic remodeling in ischemic regions. *In vivo* experiments indicate that multi-time-point continuous electroacupuncture can persistently regulate target-molecule expression in ischemic brain tissue and improve neurological deficits over time (Zhang et al., 2020). However, this study was limited to a single animal model using male Wistar rats and focused only on stimulation at Shuigou. It lacked comparisons between sexes, multi-acupoint controls, long-term follow-up of neurological function, and quantitative assessment of brain histopathology. The study also did not include drug controls, pharmacokinetic evaluation, or long-term organ toxicity assessment. Key evidence from large-animal experiments and human clinical trials is still needed before translation to stroke patients can be supported.

Existing evidence indicates that acupuncture exerts multidimensional protective effects against CIRI mainly by regulating the Wnt/β-catenin signaling pathway, including anti-apoptotic effects, promotion of angiogenesis, and neurovascular unit remodeling. However, from the perspective of translational medicine, current studies still have several notable limitations. First, mechanistic evidence largely relies on phenomenological observations of target protein expression levels, and lacks deep causal validation based on core technologies such as conditional gene knockout, as well as spatiotemporal and cell-subtype-specific analyses. Second, existing studies show marked heterogeneity in modeling methods, intervention windows, and stimulation parameters, and generally lack sham electroacupuncture controls, making it difficult to rigorously demonstrate the specificity of acupoint effects. Finally, basic evidence depends heavily on young, healthy male rodents and fails to adequately cover clinically complex pathological settings such as aging and metabolic comorbidities. Long-term neurological outcome assessment and large-animal evidence are also lacking. Future studies urgently need to incorporate aging and comorbidity models, integrate cutting-edge single-cell and spatial multi-omics technologies to precisely decode targeted regulatory network maps, and perform cross-validation using high-quality multicenter clinical cohorts and human biological samples, with the aim of constructing a complete, high-level evidence chain from basic mechanistic elucidation to precise clinical translation.

## 6 Conclusion and perspectives

CIRI is a common secondary brain injury that occurs after recanalization therapies such as intravenous rt-PA thrombolysis and mechanical thrombectomy in patients with ischemic stroke. It is also a key pathological factor that limits the benefits of thrombolysis and contributes to persistent motor and cognitive deficits. At present, clinically available neuroprotective drugs that can specifically block the cascade of CIRI remain lacking, and conventional symptomatic supportive strategies are insufficient to simultaneously control multiple injury pathways in the acute phase and initiate endogenous neural repair programs during recovery. A large body of basic experimental evidence shows that the canonical Wnt/β-catenin pathway, together with the non-canonical Wnt/PCP and Wnt/Ca^2+^ subtypes, forms a signaling regulatory network that serves as a central molecular hub for balancing CIRI progression and neurovascular unit repair. TCM has inherent advantages, including multi-component synergy, multi-target coverage, and stage-specific intervention across disease progression. TCM monomers, compound formulas, and acupuncture therapies can all target the Wnt signaling network, synchronously blocking injury cascades and activating endogenous repair programs. These characteristics help compensate for the limitations of single-target Western pharmacological interventions and support the unique research value and clinical translational potential of TCM in full-course ischemic stroke management. Ramakrishna et al., through studies involving Alzheimer’s disease, Parkinson’s disease, and other neurodegenerative disorders, showed that Wnt signaling exhibits temporal and cell-specific regulatory features across multiple central nervous system diseases, providing a broader comparative perspective for interpreting CIRI-related mechanisms (Ramakrishna et al., 2023).

Existing preclinical studies at the animal and cellular levels have systematically confirmed the efficacy of diverse TCM interventions in regulating Wnt signaling and antagonizing CIRI. Oxidative stress and inflammatory cascades induced by ischemia-reperfusion abnormally increase the activity of the GSK-3β degradation complex, accelerate ubiquitin-mediated degradation of β-catenin, and inhibit transcriptional activity of the canonical pathway. At the same time, these pathological processes disrupt non-canonical pathway-mediated regulation of cell polarity and calcium homeostasis, triggering neuronal apoptosis, BBB disruption, excessive glial activation, impaired angiogenesis, and other pathological changes. TCM monomers, including phenolic acids, flavonoids, and saponins, mainly inhibit GSK-3β function, increase Wnt ligand expression, stabilize β-catenin, and promote its nuclear translocation. These effects downregulate pro-inflammatory and pro-apoptotic molecules, promote neural stem cell proliferation, and facilitate beneficial astrocyte transdifferentiation. Compound formulas composed of multiple herbs can simultaneously balance several Wnt pathway branches, integrating vascular barrier protection in the acute phase, suppression of peripheral immune-cell infiltration into the central nervous system, and neurovascular remodeling during recovery. This pattern closely matches the dynamic pathological evolution of CIRI. Acupuncture, especially electroacupuncture, can activate Wnt signaling upstream through miRNA-mediated epigenetic mechanisms, improve microcirculation in ischemic regions, and repair the damaged neurovascular unit. Overall, different TCM interventions can regulate the crosstalk between canonical and non-canonical pathways, maintain brain-cell homeostasis and the brain-tissue microenvironment, provide experimental support for integrated Chinese and Western prevention and treatment of ischemic stroke, and open new molecular targets for post-stroke neurological remodeling.

Although current basic research has gradually clarified the molecular mechanisms by which TCM targets the Wnt network to counteract CIRI, several limitations remain and seriously hinder the translation of basic findings into precise clinical interventions. At the mechanistic level, existing studies generally emphasize the canonical pathway while paying insufficient attention to non-canonical pathways. The cooperative and antagonistic interactions between these two pathway classes have not been systematically explained. The differential regulation of Wnt signaling by TCM across brain-cell types and disease stages remains unclear, and potential targets in non-canonical pathways remain underexplored. Global research perspectives are also clearly divergent: domestic studies tend to focus on compound compatibility and multi-pathway synergy in acupuncture, whereas international studies are often limited to efficacy evaluation of single natural monomers. This pattern does not fully reflect the core holistic advantages of TCM. Clinically, multicenter, large-sample, double-blind, controlled, high-quality trials remain scarce. Few studies use Wnt pathway molecular expression as a core biomarker for efficacy evaluation, and systematic data are lacking on baseline pathway expression and drug-metabolism differences among populations. In addition, Wnt signaling has prominent time- and cell-dependent dual effects. Moderate activation in the acute phase may be protective, whereas long-term sustained activation may induce adverse outcomes such as glial scarring and cerebral tissue fibrosis. However, no unified safety threshold for pathway activation or optimal intervention window for TCM has been established. Together with unclear brain-penetrating active components of herbal medicines, low brain-delivery efficiency, lack of unified standards for acupuncture points and stimulation parameters, substantial differences between animal models and human stroke pathology, and the absence of peripheral non-invasive monitoring biomarkers, these challenges constitute the major barriers to clinical translation in this field.

Future studies should improve the research framework from three dimensions: deeper mechanistic exploration, clinical system construction, and translational technological innovation, thereby promoting practical application of TCM strategies targeting the Wnt pathway. At the basic mechanistic level, research should balance studies of canonical and non-canonical pathways, systematically clarify their interactions during the acute and recovery phases of ischemia, distinguish pathway functions specific to neurons, endothelial cells, and glial cells, identify new intervention targets in non-canonical pathways, and define the complete molecular chain through which herbal multi-component systems and acupuncture multi-route regulation synergistically modulate the Wnt network. At the clinical level, multicenter global collaboration platforms should be established to standardize TCM and acupuncture intervention protocols and pathway-biomarker detection methods. Trials combining TCM with thrombolysis or thrombectomy should be conducted to quantify its clinical value in reducing reperfusion-related complications and improving long-term cognitive and motor outcomes, while also enriching clinical data across different populations. At the translational technology level, single-cell sequencing and spatial multi-omics should be used to identify core brain-penetrating pharmacodynamic substances, brain-targeted improved delivery systems should be developed, safety evaluation systems for the degree of Wnt pathway activation and peripheral non-invasive monitoring technologies should be established, and long-term safety evaluation protocols should be refined. Ultimately, these efforts may support a staged and individualized integrated Chinese and Western medical strategy that protects the brain in the acute phase and promotes neural regeneration during recovery, providing a new theoretical basis and clinical implementation pathway for precision prevention and treatment of ischemic stroke.
